# Determination of Intracellular Esterase Activity Using Ratiometric Raman Sensing and Spectral Phasor Analysis

**DOI:** 10.1021/acs.analchem.2c05708

**Published:** 2023-03-16

**Authors:** Henry J. Braddick, William J. Tipping, Liam T. Wilson, Harry S. Jaconelli, Emma K. Grant, Karen Faulds, Duncan Graham, Nicholas C. O. Tomkinson

**Affiliations:** † Department of Pure and Applied Chemistry, Thomas Graham Building, University of Strathclyde, 295 Cathedral Street, Glasgow G1 1XL, U.K.; ‡ Centre for Molecular Nanometrology, Department of Pure and Applied Chemistry, Technology and Innovation Centre, University of Strathclyde, 99 George Street, Glasgow G1 1RD, U.K.; § GlaxoSmithKline Medicines Research Centre, Gunnels Wood Road, Stevenage SG1 2NY, U.K.

## Abstract

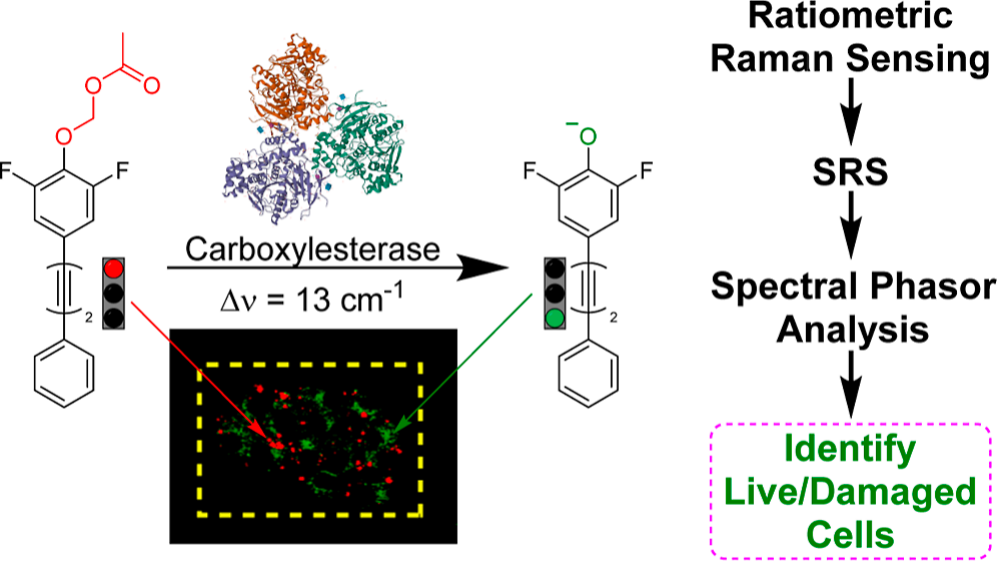

Carboxylesterases (CEs) are a class of enzymes that catalyze the hydrolysis of esters in a variety of endogenous and exogenous molecules. CEs play an important role in drug metabolism, in the onset and progression of disease, and can be harnessed for prodrug activation strategies. As such, the regulation of CEs is an important clinical and pharmaceutical consideration. Here, we report the first ratiometric sensor for CE activity using Raman spectroscopy based on a bisarylbutadiyne scaffold. The sensor was shown to be highly sensitive and specific for CE detection and had low cellular cytotoxicity. In hepatocyte cells, the ratiometric detection of esterase activity was possible, and the result was validated by multimodal imaging with standard viability stains used for fluorescence microscopy within the same cell population. In addition, we show that the detection of localized ultraviolet damage in a mixed cell population was possible using stimulated Raman scattering microscopy coupled with spectral phasor analysis. This sensor demonstrates the practical advantages of low molecular weight sensors that are detected using ratiometric Raman imaging and will have applications in drug discovery and biomedical research.

## Introduction

Carboxylesterases (CEs) are a ubiquitous class of enzymes within the esterase family that hydrolyze exogenous and endogenous carboxylesters to their corresponding carboxylic acids.^1^ CEs can be divided into five major groups (CE1–CE5), with the majority falling into the CE1 or CE2 families.^2^ In mammals, liver cells, which play a primary role in metabolism, display the highest levels of CE activity.^2^ Aberrant CE activity has been directly linked to numerous diseases including obesity,^3^ cancer,^4^ and hepatic steatosis,^3^ and therefore, sensors for the detection of esterase activity are important tools for the study of drug metabolism and disease progression. Although the use of fluorescence microscopy for sensing intracellular esterase has been well established,^5^ the inherent “on/off” nature and concentration dependency of many fluorescent sensors makes ratiometric analyses difficult, and the broad linewidth of fluorescent emission signals (∼1500 cm^–1^) results in a color barrier that can prevent multiplex analysis of different intracellular targets.^6^ In addition, the use of fluorescent probes in live cells and tissues has been impractical due to the short excitation wavelengths (<400 nm) required for some scaffolds, which result in photodamage and short tissue penetrating depth,^7^ while the photobleaching of these probes can render repeat analysis impossible. A promising esterase sensor based on two-photon excitation has been recently reported, which overcomes many of these limitations.^8^ However, the complex scaffold requires either long synthetic routes or expensive starting materials to prepare, limiting accessibility.

Raman microscopy is a powerful tool for the non-destructive visualization of biomolecules, cells, and tissues.^9^ Vibrational-tag Raman imaging has enabled the study of the intracellular interactions of a variety of exogenous probes, with the flagship method being alkyne-tag Raman imaging (ATRI).^10^ Alkyne groups exhibit a strong vibration within the cell-silent region of a Raman spectrum (1800–2800 cm^–1^), which allows for their straightforward detection within biological samples.^11^ Since the initial application of ATRI in the study of nucleic acids,^12^ the technique has been used to visualize the metabolism and distribution of proteins,^13,14^ lipids,^13−15^ and drug molecules,^16−19^ among other species.^20^

**Figure 1:**
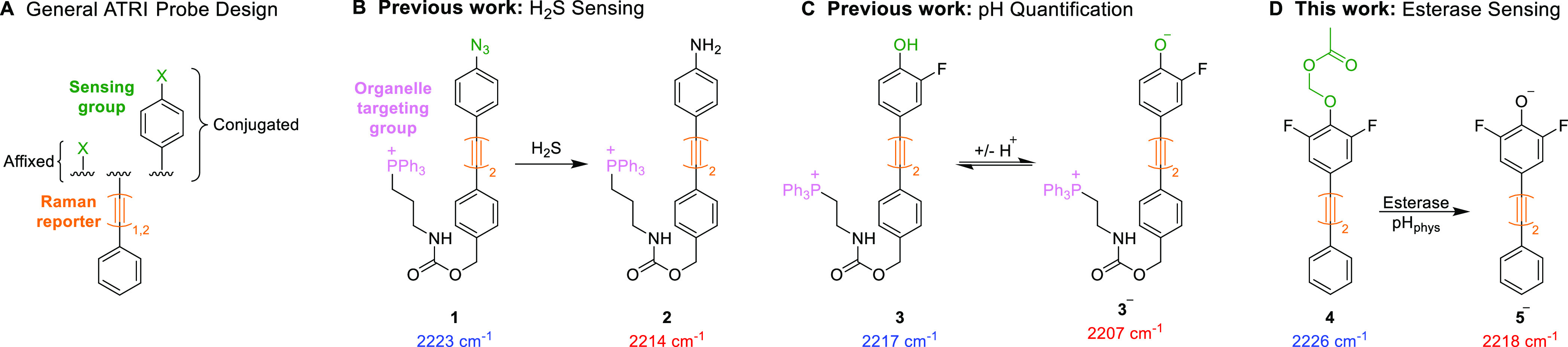
Examples of intracellular sensing using ATRI. (A) General structure of ATRI-based sensors. (B) Bisarylbutadiyne sensor for hydrogen sulfide **1**.^23^ (C) Reversible sensor for the quantification of pH **3**.^26^ (D) Detection of intracellular esterase activity using sensor **4**.

The narrow linewidth of Raman bands (<20 cm^–1^) has enabled the development of Raman sensors. Recent examples have included the detection of ionic species,^21,22^ intracellular hydrogen sulfide,^23^ and pH (Figure 1).^24−26^ In each case, probe molecules contain a suitably reactive sensing group in conjugation with, or affixed to, an alkyne or nitrile moiety (Figure 1A), and reaction of the sensing group with the analyte of interest yields a change in the vibrational properties of the sensor. Ratiometric sensors of this nature are advantageous due to the intrinsic referencing ability and inherent quantification benefits that accompany ratiometric methods.^27^

Vibrational-tag Raman imaging has previously been applied to enzyme sensing as an alternative approach to multiplex detection, with Fujioka et al. simultaneously detecting four unique enzymes using electronic pre-resonance stimulated Raman scattering (EPR–SRS).^6^ Xanthene derivatives targeted to different enzyme substrates, together with isotopic editing (^12^C/^13^C and ^14^N/^15^N) of a conjugated nitrile moiety, enabled the specific detection of each enzyme simultaneously at discrete wavenumbers. The Raman sensors were activated when the molecular absorption of the xanthene core was shifted from the visible (electronic non-resonant condition) to the near infrared (NIR and EPR condition) upon reaction with the target biomolecule.^28^

Herein, we describe the first ratiometric Raman sensor for intracellular imaging of esterase activity using SRS microscopy. Synthesized using an accessible strategy, **4** is a low-molecular-weight (<350 Da) bisarylbutadiyne probe that is detected using NIR irradiation. The sensor contains an acetoxymethyl (AM) group, which, upon cleavage, yields an acidic phenol group that results in a red-shifting of the diyne stretching frequency at physiological pH. Our method allows for the sensitive and selective detection of esterase enzyme activity and represents an adaptable strategy for the sensing of different enzyme classes. Finally, we show that regions of damage within a cell population can be identified using spectral phasor analysis within a single experiment, providing a novel platform to assess cell viability.

## Experimental Section

### Procedure for Spontaneous Raman Spectroscopy Mapping Experiments

Cells were plated on glass-bottomed culture dishes (35 mm high, Ibidi) at a concentration of 5 × 10^5^ cells per well and incubated at 5% CO_2_ and 37 °C for 24 h prior to compound treatment. For live cell imaging, cells were treated with compound **4**, **5**, or **6** (10 μM, diluted from a 20 mM stock solution in DMSO) in media and incubated at 5% CO_2_ and 37 °C for 30 min. The dishes were then aspirated and washed with PBS (3 × 2 mL) before the cells were imaged in PBS. To simulate dead cells, cells were pre-treated with PFA (4% v/v) and Triton X-100 (0.05% v/v) in PBS for 2 h before being washed with PBS (3 × 2 mL), treated with **4**, **5**, or **6** (10 μM, diluted from a 20 mM stock solution in DMSO) in media, and incubated at 5% CO_2_ and 37 °C for 30 min. The dishes were then aspirated and washed with PBS (3 × 2 mL) before imaging in PBS. Raman maps were acquired using λ_ex_ = 532 nm with a Nikon 60×/NA 1.0 NIR Apo water immersion objective, 5 μm step size in *x* and *y*, 0.5 s acquisition time, a laser power of 100% (36 mW), and a spectral center of 2800 cm^–1^. Three replicate maps were acquired from different culture plates for each condition. Average spectra were calculated for each cell map in MatLab R2022a, from which intensity ratios at 2212 and 2226 cm^–1^ were extracted.

### General Procedure for SRS Imaging Experiments

Cells were plated in six-well plates containing high-precision glass coverslips (#1.5 H, 22 × 22 mm; Thorlabs) at a concentration of 5 × 10^5^ cells per well and incubated in media at 5% CO_2_ and 37 °C for 24 h prior to compound treatment. For live cell imaging, cells were treated with **4** or **5** (10 μM, diluted from a 20 mM stock solution in DMSO) in media and incubated at 5% CO_2_ and 37 °C for 30 min. The wells were then aspirated and washed with PBS (3 × 2 mL). The coverslips were then removed from the wells and affixed to microscope slides for imaging with a PBS boundary. To simulate dead cells, cells were pre-treated with PFA (4% v/v) and Triton X-100 (0.05% v/v) in PBS for 2 h. The wells were then aspirated and washed with PBS (3 × 2 mL), treated with **4** or **5** (10 μM, diluted from a 20 mM stock solution in DMSO) in media, and incubated at 5% CO_2_ and 37 °C for 30 min. The wells were then aspirated and washed with PBS (3 × 2 mL). The coverslips were then removed from the wells and affixed to microscope slides for imaging with a PBS boundary (see the Supporting Information for full experimental details).

## Results

**Figure 2:**
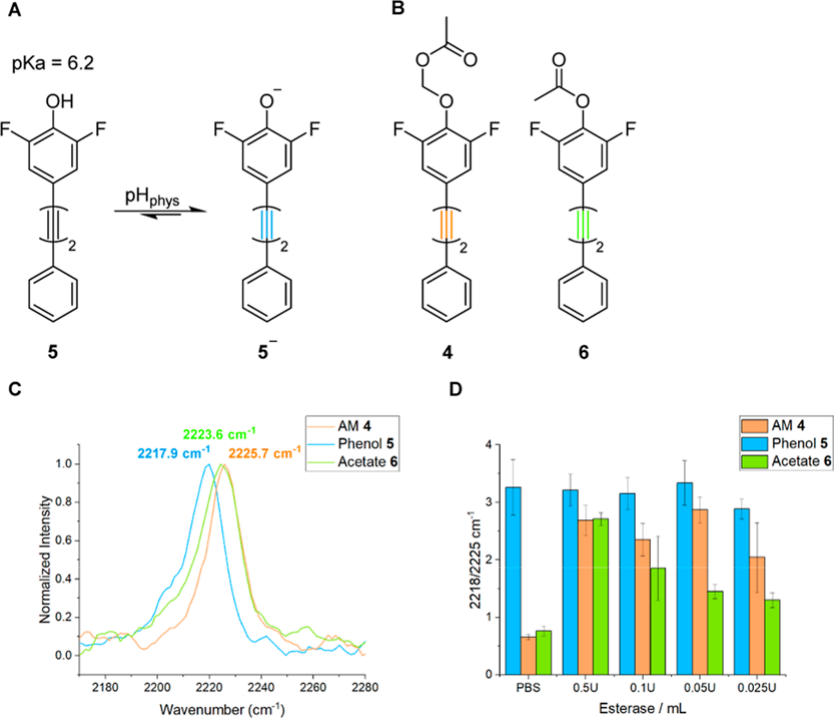
Development of a bisarylbutadiyne esterase sensor. (A) Deprotonation of the difluorophenol scaffold **5** at physiological pH. (B) Esterase-sensitive compounds **4** and **6** synthesized as part of this work. (C) Overlaid Raman alkyne peaks of difluorophenol **5** (blue), AM ester **4** (orange), and acetate **6** (green) [100 μM, PBS/DMSO (pH 7.4, 8:2 v/v), 532 nm, 1 × 20 s exposure, 50× lens. Spectra were acquired after 1 h of incubation at 37 °C]. Peak centers were determined using a non-linear Gauss fitting function (Orgin2021). (D) LoD study of esters **4** and **6** using PLE (100 μM, PBS/DMSO (8:2 v/v), 532 nm, 1 × 20 s exposure, 50× lens. Spectra were acquired after 1 h of incubation at 37 °C).

For a Raman-based probe to fully benefit from ratiometric sensing, the Raman peak of interest must undergo a discernible spectroscopic shift (>7.5 cm^–1^) following interaction with the analyte. Previous work has shown that in the case of a bisarylbutadiyne scaffold, it was possible to induce a large Raman alkyne shift (Δν_alkyne_) by introducing a formal charge in conjugation with the oligoyne chain.^25^ This phenomenon was exploited to generate a library of pH sensors with a range of p*K*_a_ values (2–10), and we postulated that this concept could be applied to enzymatic sensing. We envisaged an esterase sensitive probe that, upon reaction with an enzyme, liberated a compound that was ionized under physiological conditions (37 °C, pH 7.4) and thereby induced a significant Raman alkyne shift. Therefore, difluorophenol **5** (Figure 2A) was selected as the scaffold for our sensor. With a p*K*_a_ of 6.2, the phenol group is deprotonated at physiological pH, forming the conjugate base **5**^**–**^.^25^ We targeted the corresponding acetate (**6**) and AM ester (**4**) (Figure 2B), which are effective and stable esterase-sensitive head groups in “pro-fluorophore” approaches to sensing intracellular esterase activity.^8,29,30^ Compounds **4** and **6** were prepared from commercial starting materials through four-step syntheses (see the Supporting Information for full synthetic procedures).

The efficacies of **4** and **6** as esterase sensors were assessed by comparing their sensitivity, selectivity, and Δν_alkyne_ upon incubation with the commercially available mammalian esterase, porcine liver esterase (PLE). First, it was deemed that a larger Δν_alkyne_ value between the probe molecule and **5**^**–**^ was desirable in order to facilitate ratiometric sensing. Compounds **4**–**6** were analyzed in a mixture of PBS (pH 7.4) and DMSO (8:2 v/v), and the alkyne peak centers were determined (Figure 2C). The AM ester **4** showed a greater Δν_alkyne_ value than that of acetate **6**, with values of 7.8 and 5.7 cm^–1^, respectively, indicating the potential of **4** to function as a ratiometric sensor. The in vitro enzymatic reactivity of both compounds was then assessed using PLE (Figure 2D). The hydrolysis of each ester in the presence of PLE was deduced using the ratio of the signal intensities at 2218 and 2225 cm^–1^, and the AM ester **4** was identified as the more effective esterase substrate due to its lower limit of detection toward PLE. Partial conversion of **4** to **5** was observed using only 0.025 U/mL of PLE after 1 h of incubation, providing a promising esterase probe with a large Δν_alkyne_ value and high sensitivity, comparable to a recent fluorescent esterase sensor.^31^ As a control experiment, PLE was denatured by heating at 90 °C for 3 h prior to the addition of **4** (100 μM, 30 min). In this case, no hydrolysis of **4** to **5** was observed (Figure S1). The reactivity of **4** and **6** toward PLE over a 90 min period was also assessed, with both compounds found to hydrolyze to phenol **5** at similar rates (Figure S2). We assessed the specificity of both sensors by incubating each compound with a variety of amino acids, salts, and biomolecules. AM ester **4** was found to be more stable than acetate **6** in the presence of the interference agents used (Figure S3A/S3B), demonstrating the specificity of **4** toward esterase-catalyzed hydrolysis. In addition, the pH stability of **4** was investigated by dissolving the probe in Britton–Robinson buffers at fixed pH values of 5.31 and 9.43 and PBS (pH 7.4) (Figure S4). Each solution was analyzed repeatedly over a 2 h period at room temperature using spontaneous Raman spectroscopy. In each case, no conversion to phenol **5** was observed. In addition, **4** showed no photodegradation over the same time period. Finally, the cytotoxicity of esters **4** and **6** and phenol **5** was investigated against HepG2 cells, with all compounds found to have no effect on cell viability after incubation at up to 20 μM for 8 h (Figure S5). These results were consistent with other bisarylbutadiyne compounds of this nature and demonstrated the suitability of **4**, **5**, and **6** for cell-based studies.^25^ While the precise reasons for its superior performance are unknown, based on the higher sensitivity, stability, and larger Δν_alkyne_ of **4**, this compound was taken forward for cellular studies.

**Figure 3:**
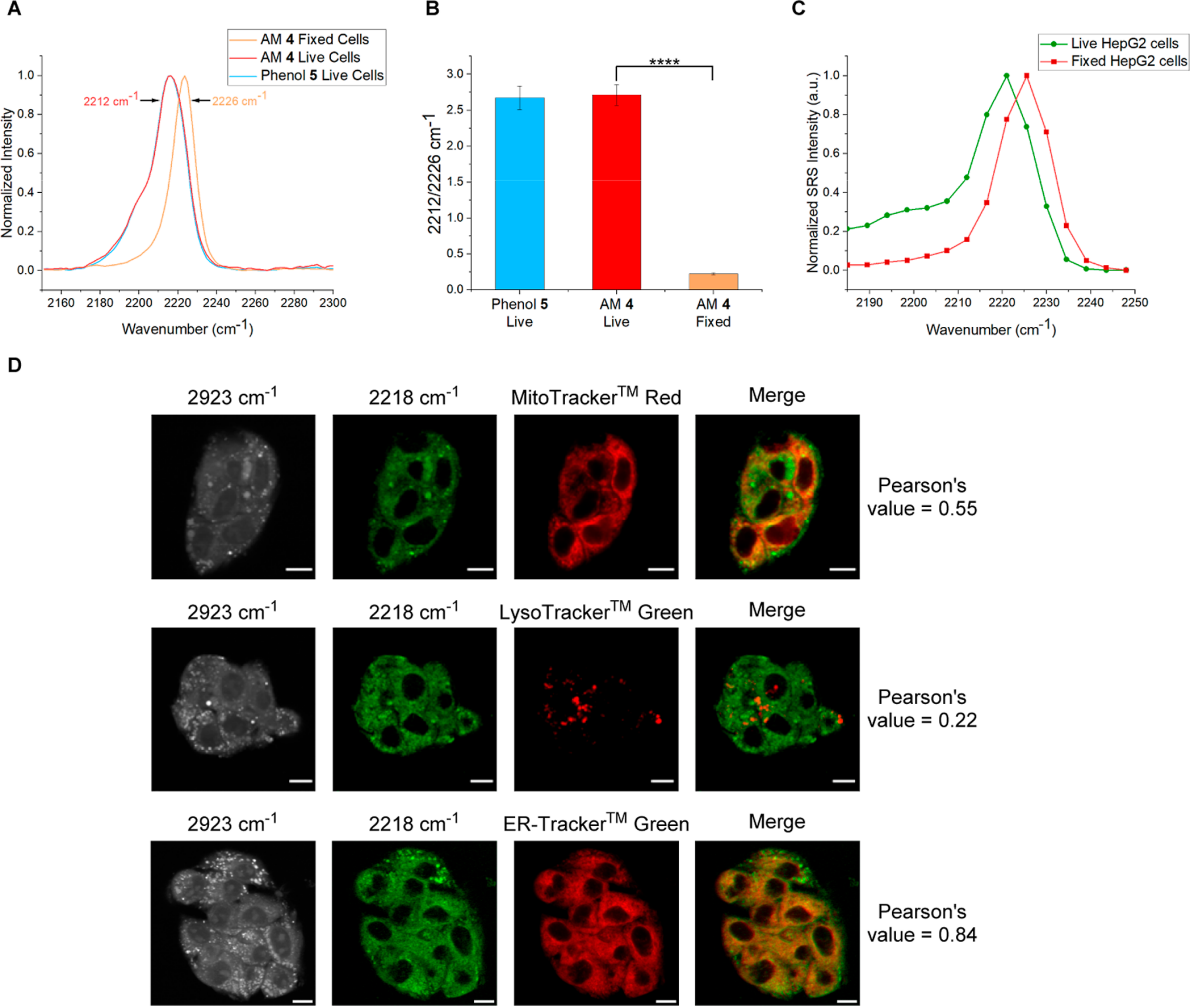
Assessment of **4** as an intracellular esterase sensor. (A) Overlaid alkyne peaks of the average spectra of difluorophenol **5** in live HepG2 cells (blue), AM ester **4** in live HepG2 cells (red), and **4** in fixed HepG2 cells (orange). [532 nm, 1 × 0.5 s exposure, 60× lens, 1 μm step size. Maps were acquired after treatment with **5** or **4** (10 μM) in media for 30 min. To fix, cells were pre-treated with PFA (4% v/v) and Triton X-100 (0.05% v/v) in PBS for 2 h prior to addition of **5** or **4**]. (B) Ratio of peak intensities at 2212 and 2226 cm^–1^ taken from the average spectra of maps of difluorophenol **5** in live HepG2 cells and AM ester **4** in live HepG2 cells or fixed HepG2 cells. [532 nm, 1 × 0.5 s exposure, 60× lens, 5 μm step size. Maps were acquired after treatment with **5** or **4** (10 μM) in media for 30 min. To fix, cells were pre-treated with PFA (4% v/v) and Triton X-100 (0.05% v/v) in PBS for 2 h prior to addition of **5** or **4**]. ****T test *p* ≤ 1 × 10^–4^. (C) Pseudo-Raman spectra generated from SRS spectral sweeps (2248–2185 cm^–1^, 14 images) of **4** in live HepG2 cells and in fixed HepG2 cells. All images were acquired at 512 × 512 pixels and a 9–48 μs pixel dwell time. Images were acquired after treatment with **4** (10 μM) in media for 30 min. To fix, cells were pre-treated with PFA (4% v/v) and Triton X-100 (0.05% v/v) for 2 h prior to addition of **4**. (D) Tandem SRS–fluorescence imaging of live HepG2 cells treated with a solution of **4** (10 μM) and appropriate working concentrations of organelle stains (MitoTracker red 250 nM; LysoTracker green 62.5 nM; ER-Tracker green 1 μM) in media. Fluorescence images were acquired initially (MitoTracker red λ_ex_ = 633 nm, λ_em_ = 640–750 nm; LysoTracker green λ_ex_ = 488 nm, λ_em_ = 495–600 nm; ER-Tracker green λ_ex_ = 488 nm, λ_em_ = 495–600 nm) before SRS images at 2923 cm^–1^ (CH_3_, protein) and 2218 cm^–1^ (alkyne). All images were acquired at 512 × 512 pixels and a 9–48 μs pixel dwell time. False colors and scale bars representing 10 μm were applied in ImageJ. Merged images of **4** and the organelle stains were generated in ImageJ and the Pearson’s R values were calculated using the Coloc2 tool.

The efficacy of **4** for intracellular esterase sensing was next assessed (Figure 3). We selected HepG2 (hepatocellular carcinoma) cells for the analysis of our sensor due to the high level of CEs present in mammalian hepatocytes.^2^ Live HepG2 cells were treated with **4** (10 μM, 30 min), and the average Raman spectrum was plotted from the mapping data acquired (Figure 3A). In live cells, the alkyne Raman shift was detected at 2215.1 cm^–1^, which was concordant with difluorophenol **5** and suggested that the expected ester hydrolysis had occurred after just 30 min of treatment. To validate this result, dead HepG2 cells were simulated by fixing with paraformaldehyde (PFA, 4% v/v) and Triton X-100 (0.05% v/v) in PBS and were then treated with **4** (10 μM, 30 min). In these cells, the observed Raman alkyne shift was 2222.8 cm^–1^, indicating an absence of esterase activity likely due to the denaturation of cellular proteins.

When the spectra of **4** in live and fixed cells are overlaid, it becomes apparent that the ratiometric ability of the probe can be further enhanced by analyzing the Raman intensity at wavenumber values located on the shoulders of the peaks of interest. This approach has previously been shown to facilitate ratiometric measurements using SRS microscopy.^23^ An increase in Δν_alkyne_ to 14 cm^–1^ could be achieved by adopting this strategy and measuring the 2212 cm^–1^/2226 cm^–1^ signal intensity ratio. To demonstrate this, the ratio of the Raman signal intensities at 2212 and 2226 cm^–1^ was extracted from three different mapping repeats of **4** in live and fixed cells and compared to the ratio values of phenol **5** in live cells, which was used as a control (Figure 3B). In live cells treated with **4**, the ratio 2212/2226 cm^–1^ was ∼2.5, consistent with the ratio observed in live cells treated with phenol **5**. The ratio was also determined in fixed cells treated with **4** and was found to be ∼0.3, significantly different to the live cell sample, indicating a clear potential for differentiating live/fixed cell populations using probe **4**.

After demonstrating the ability of **4** to act as a ratiometric esterase sensor with a suitable Δν_alkyne_ value using spontaneous Raman spectroscopy, we sought to use SRS for the high-resolution visualization of esterase activity within cells. Live and fixed HepG2 cells were treated with **4** (10 μM, 30 min) before imaging with SRS microscopy. Pseudo-Raman spectra were generated from SRS spectral sweeps between 2248 and 2185 cm^–1^ from the live and fixed HepG2 populations, and the overlaid spectra indicated that shoulder analysis was also possible with SRS (Figure 3C), albeit with blue-shifted wavenumber values when compared to spontaneous Raman spectroscopy due to an inherent offset within the SRS equipment. This effect has been observed in previous work.^26^ Analysis of Figure 3C indicated that 2232 and 2219 cm^–1^ represented suitable wavenumber values for the ratiometric shoulder analysis of **4** and the corresponding phenolate **5**^**–**^ after esterase-catalyzed hydrolysis.

We next sought to determine the intracellular localization of **4**. Figure 3D shows false-color tandem SRS–fluorescence microscopy images of HepG2 cells treated with **4** (10 μM, 30 min) and different organelle stains (MitoTracker red, 250 nM; LysoTracker green, 62.5 nM; ER-Tracker green, 1 μM, each 30 min). Imaging at 2923 and 2218 cm^–1^ enabled the visualization of intracellular protein and the distribution of **4** within the populations, respectively. Co-localization analyses of the signal at 2218 cm^–1^ and the fluorescence signal of the organelle stains revealed that our sensor strongly localizes to the endoplasmic reticulum, with a Pearson’s *R* value of 0.84, as expected for lipophilic compounds such as **4**.^32^ It was also found that the distribution of **4** poorly correlated to the lysosomal (*R* = 0.22) and mitochondrial (*R* = 0.55) compartments.

**Figure 4:**
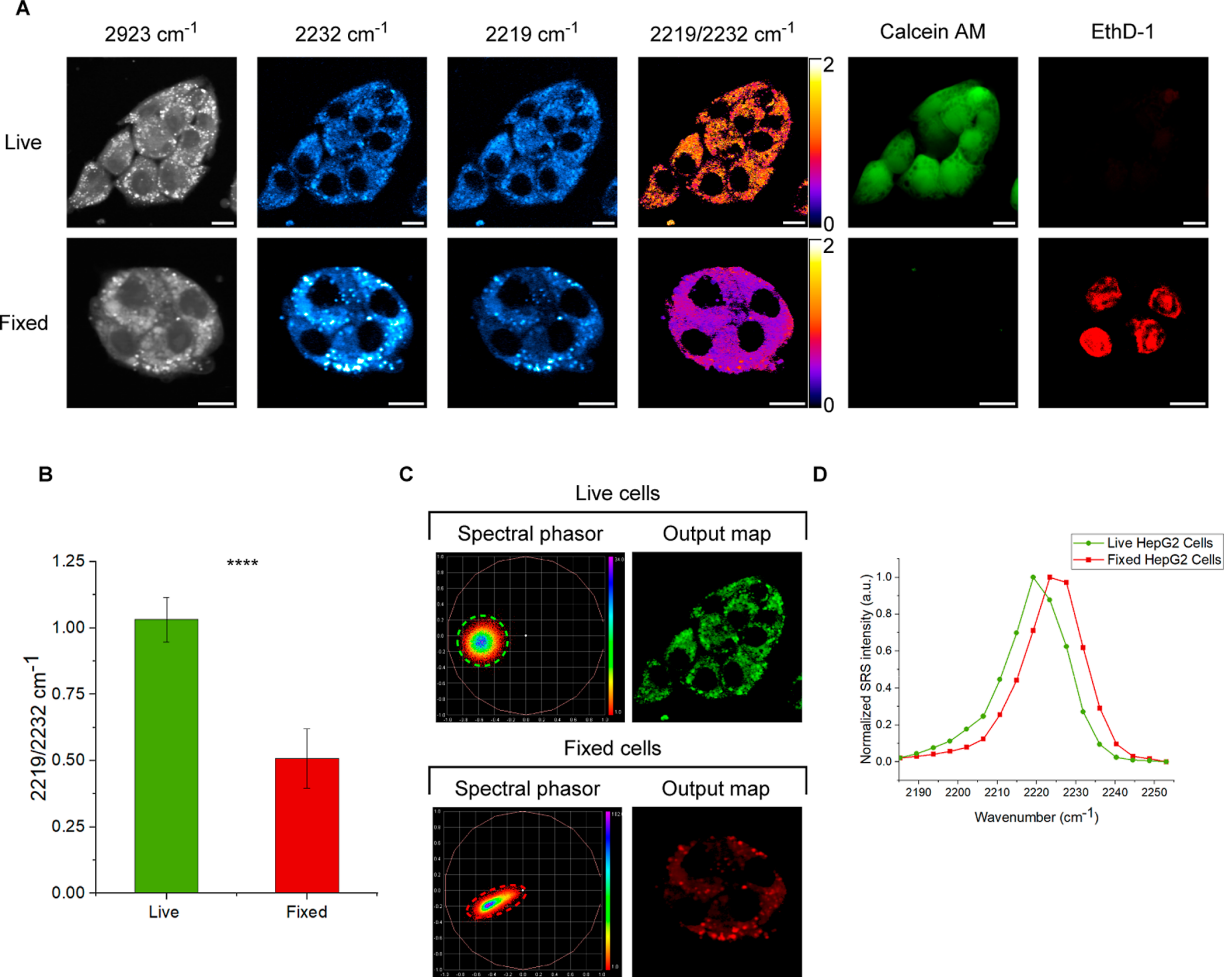
Ratiometric and phasor analysis of **4** as an intracellular esterase sensor. (A) Ratiometric study of **4** in live and fixed HepG2 cells treated with cell viability stains ethidium homodimer (EthD-1) and calcein AM [to fix, cells were pre-treated with PFA (4% v/v) and Triton X-100 (0.05% v/v) in PBS for 2 h prior to addition of **4** and cell viability stains]. Images were acquired after treatment with **4** (10 μM), EthD-1 (4 μM), and calcein AM (2 μM) in media for 30 min. Fluorescence images were acquired initially (EthD-1 λ_ex_ = 514 nm, λ_em_ = 540–650 nm; calcein AM λ_ex_ = 488 nm, λ_em_ = 493–526 nm) before SRS images at 2923 cm^–1^ (CH_3_, protein) and SRS spectral sweeps (2253–2181 cm^–1^, 18 images). Images at 2232 and 2219 cm^–1^ were taken from the corresponding images of the SRS spectral sweeps. All images were acquired at 512 × 512 pixels, 9–48 μs pixel dwell time. False colors and scale bars representing 10 μm were applied in ImageJ. Ratio bars show the Fire LUT scaled between values of 0 and 2. (B) Ratio of the intensities at 2219 and 2232 cm^–1^ in live and fixed HepG2 cells. Pseudo-Raman spectra were generated from >3 cells in each spectral sweep (2253–2181 cm^–1^, 18 images), and the intensities at 2219 and 2232 cm^–1^ were extracted. ****T test *p* ≤ 1 × 10^–4^. (C) Spectral phasor analysis of the SRS spectral sweeps (2253–2181 cm^–1^, 18 images) of live and fixed HepG2 cells treated with **4** as seen in (A). SRS spectral sweeps were background-subtracted on ImageJ, and phasor plots were generated using an ImageJ plugin. The corresponding images of live and fixed cells were then generated from appropriate ROIs on the spectral phasor plot. (D) Overlaid pseudo-Raman spectra of the live and fixed HepG2 cells taken from the spectral phasor output maps.

An advantage of Raman-based imaging is that it is compatible with other imaging modalities including fluorescence. To demonstrate the ability of **4** to act as an intracellular esterase sensor using SRS and as a means of determining cell viability, live and fixed HepG2 cells were treated with **4** (10 μM, 30 min) and the cell viability stains ethidium homodimer (EthD-1, 4 μM, 30 min) and calcein AM (2 μM, 30 min) (Figure 4A). The ethidium homodimer is a DNA-binding, membrane-impermeable stain used to visualize dead cells, while calcein AM is a pro-fluorophore that acts as a live cell stain following an esterase-mediated hydrolysis to activate the fluorophore. SRS spectral sweeps between 2253 and 2181 cm^–1^ enabled the ratiometric comparison of signal intensities at 2232 and 2219 cm^–1^. These wavenumber values were chosen as they displayed the greatest difference in ratio between live and fixed cells, therefore best facilitating ratiometric esterase sensing. In live cells, as confirmed by a positive calcein AM fluorescent signal and a lack of signal in the EthD-1 channel, the ratio of the signal intensities at 2219 and 2232 cm^–1^ across a number of cells (>3) was 1.03 ± 0.08. In contrast, cells fixed with PFA (4% v/v) and Triton X-100 (0.05% v/v) showed a fluorescent signal arising from EthD-1 and an absence of signal in the calcein AM channel, confirming that the cells were not viable and the ratio of the signal intensities at 2219 and 2232 cm^–1^ was significantly different to the live cell value, with a value of 0.51 ± 0.11 (Figure 4B). As a control experiment, live and fixed HepG2 cells were treated with phenol **5** (10 μM, 30 min), and in each case, the observed 2219/2232 cm^–1^ ratio was ∼1.09, consistent with the value from **4** in live cells (Figure S6). To demonstrate the applicability of the ratiometric sensor, detection in a series of cell lines (HeLa, U-87, and SK-BR-3) was performed (Figure S7). In each case, the ratio of the signal intensities at 2219 and 2232 cm^–1^ in live and fixed populations was significantly different, consistent with our findings in HepG2 cells. As such, **4** represents an effective tool for the determination of cell viability and ratiometric sensing of esterase enzyme activity across a range of cell lines, facilitated by the sensitivity, stability, and spectroscopic profile of the compound.

Spectral phasor analysis of SRS images is a powerful technique for cellular segmentation based directly on the SRS spectrum at each pixel location within the image. Pioneered by Fu et al.,^33^ it has recently been applied to monitoring intracellular lipid abundance in response to treatment with statins and for SRS-based imaging cytometry.^34,35^ Hyperspectral SRS data can be processed with spectral phasor analysis to form a phasor plot; a two-dimensional map consisting of spectral phasor data points. Each spectral phasor represents a unique Raman spectrum from within the 3D input SRS data set (with axes of *xy*λ), and the proximity of spectral phasors to one another on the phasor plot gives an indication as to the spectroscopic similarity of the input data points. Regions of the phasor plot containing tightly clustered spectral phasors can then be mapped to visualize segmented regions of the original data that possess similar Raman spectra.^33,34^ To demonstrate the application of **4** to studying mixed cell populations, we applied spectral phasor analysis to the SRS spectral sweeps (2253–2181 cm^–1^, 18 images) of **4** within live and fixed HepG2 cells (Figure 4C). We observed that SRS images of **4** in live and fixed cells occupy unique and different regions of the phasor plot as a result of the Δν_alkyne_ between **4** and phenolate **5**^**–**^ that is formed upon esterase-mediated hydrolysis. To validate this analysis, pseudo-Raman spectra of the live and fixed output maps were generated and overlaid (Figure 4D). It was found that these pseudo-Raman spectra mimic the original spectra generated from the raw SRS images of **4** in live and fixed HepG2 cells, thus confirming the suitability of a spectral phasor approach for the determination of esterase activity. Further, SRS spectral sweeps in the high wavenumber region (3050–2803 cm^–1^, 40 images) of live and fixed cells treated with **4** (10 μM, 30 min) and subsequent spectral phasor analysis enabled visualization of various cellular components (Figure S8). The SRS sweeps of live and fixed cells occupy similar regions of the phasor plot, resulting in output images displaying minimal differences in the cellular structure of live and fixed cells, thereby confirming that the observations in Figure 4C arise from **4** and its hydrolysis in live cells.

**Figure 5:**
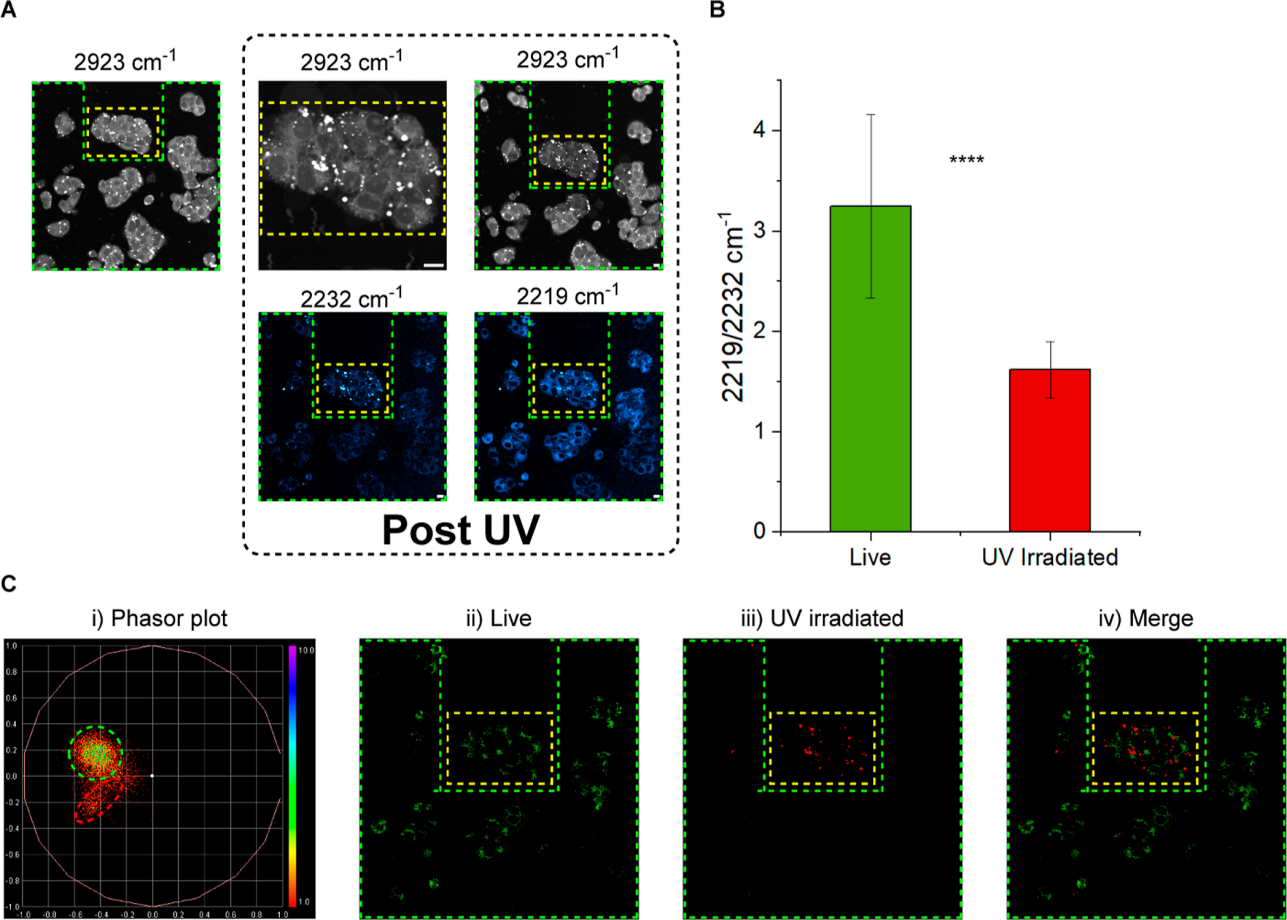
Localized UV irradiation experiment and subsequent phasor analysis. (A) Study of **4** in live and UV-irradiated cells. Following UV irradiation, images were acquired after treatment with **4** (10 μM) in media for 30 min. Images at 2232 and 2219 cm^–1^ were taken from the corresponding images of SRS spectral sweeps (2253–2181 cm^–1^, 18 images). All images were acquired at 512 × 512 pixels, 9–48 μs pixel dwell time. False colors and scale bars representing 10 μm were applied in ImageJ. (B) Ratio of the intensities at 2219 and 2232 cm^–1^ in live and UV-irradiated HepG2 cells. Pseudo-Raman spectra were generated from >3 cells in each of the live and UV-irradiated areas of the spectral sweep (2253–2181 cm^–1^, 18 images), and the intensities at 2219 and 2232 cm^–1^ were extracted. ****T test *p* ≤ 1 × 10^–4^. (C) Spectral phasor analysis of the SRS spectral sweep (2253–2181 cm^–1^, 18 images) of live and UV-irradiated HepG2 cells as seen in (A). The SRS spectral sweep was background-subtracted on ImageJ, and a phasor plot was generated using an ImageJ plugin. The corresponding images of live and UV-irradiated cells were then generated from appropriate ROIs on the spectral phasor plot (using Figure 4C as the reference).

Having demonstrated the applicability of spectral phasor analysis for investigating single-cell populations, we aimed to demonstrate this application in mixed cell populations as a means of simultaneously visualizing the active and denatured esterase enzyme (Figure 5). To stimulate localized UV damage, we selected a small group of HepG2 cells (yellow dashed marker) within a live population on a perfusion chamber, which were irradiated with UV light (405 nm, ∼5 mW laser power, 40 min). The population of cells was then treated with **4** (10 μM, in media) and incubated at 37 °C for 10 min. SRS imaging at 2923 cm^–1^ revealed blebbing of the UV-irradiated cells, an effect associated with cell death (Figure S10).^36,37^ An SRS spectral sweep (2253–2181 cm^–1^, 18 images) allowed comparison of the signal intensities at 2219 and 2232 cm^–1^ between live and UV-irradiated cells. We observed that the signal intensity at 2232 cm^–1^ was greatest in the UV-irradiated cells, indicating that these cells contained the greatest proportion of intact AM ester **4** compared to the non-irradiated cells (green dashed marker), which possessed a greater signal intensity at 2219 cm^–1^. We compared the ratio of the intensities at 2232 and 2219 cm^–1^ in live and UV-irradiated cells and saw a significant difference between the two groups of cells (Figure 5B). The 2219/2232 cm^–1^ ratio in live cells was 3.25 ± 0.92, and the same ratio in UV-irradiated cells was 1.62 ± 0.28. The significant difference between these ratios demonstrates the disabling effect UV irradiation has on intracellular enzymatic activity, as evidenced by others.^31^ We also noted that for both the live and UV-irradiated cells, this ratio was greater than we had seen in our previous analyses (Figure 4B). This was attributed to a shift in the peak center (and subsequently the phasor plots) due to imaging these cells under physiological conditions (37 °C, in media), where previously, the images were captured at room temperature in PBS.

Finally, we applied a spectral phasor analysis to the SRS spectral sweep of the whole field of view (FOV) containing live and UV-irradiated cells (Figure 5C). The resulting phasor plot contained regions that are characteristic of both live and fixed cells as identified in the phasor plots acquired from the single-cell populations presented in Figure 4C. Selecting a region of interest (ROI) within the “live region” of the phasor plot (Figure 5Ci, green dashed marker) resulted in a segmented spectral image that was localized to the non-UV-irradiated cells as expected but also displayed regions within the UV-irradiated cells, suggesting that these regions still contained the active esterase enzyme that had successfully hydrolyzed **4** to phenol **5**. Further, by selecting an ROI within the phasor region associated with fixed cells (Figure 5Ci, red dashed marker), it was seen that the distribution of **4** is confined largely to the UV-irradiated cells. These observations suggest that after 40 min of UV irradiation, the cells are severely damaged (as evidenced by blebbing and reduced esterase activity) but still possess metabolically active regions containing the functional esterase enzyme. Using SRS, both live and damaged cells can be studied within the same FOV. Our findings highlight the potential of the use of **4** in conjunction with SRS and spectral phasor analysis to study these different cell types in the same experiment.

## Conclusions

We have reported the synthesis and application of the first low-molecular-weight (<350 Da), ratiometric esterase sensor for detection using spontaneous Raman spectroscopy and SRS microscopy. The synthetically accessible AM ester **4** is a highly selective, pH-stable, and non-cytotoxic probe for the sensing of intracellular esterase. A clear advantage of **4** compared to similar fluorescent sensors is the ratiometric output it provides, enabling the detection of the probe before and after interaction with the esterase enzyme. As such, unlike commercial live/dead stains based on mixtures of EthD-1 and calcein AM, **4** is self-referencing, meaning that a single probe is required for assessing cell viability, and ratiometric analyses are possible independent of the probe concentration. This offers further potential for multiplexing with other Raman and/or fluorescent probes for the simultaneous sensing of other intracellular species. After determining the localization of **4** within the endoplasmic reticulum of HepG2 cells, we showed that live and localized UV-damaged regions of cells could be simultaneously visualized by SRS and spectral phasor analysis. Due to the lower p*K*_a_ of **5** relative to physiological pH, the general structure of **4** represents an exciting scaffold for the sensing of alternative enzyme classes through modular design of the enzyme-sensitive group. Further, the narrow Raman linewidths exhibited by **5** and **4** hold obvious potential for the multiplex analysis of **4** with other enzyme sensors through ^13^C labeling of the alkyne groups to generate analogous enzymatic probes.

## Data Availability

The raw data supporting this research publication will be made available from the University of Strathclyde at the following link: https://pureportal.strath.ac.uk/en/datasets/data-for-determination-of-intracellular-esterase-activity-using-r.

## Supporting Information Available

Experimental procedures for all experiments presented in this article; data pertaining to the imaging and analysis of **4**, **5**, and **6**; experimental procedures; and ^1^H, ^19^F, and ^13^C NMR spectra and other analytical data for all compounds reported in the manuscript (PDF)
