# Supplementary material for: Determination of Intracellular Esterase Activity Using Ratiometric Raman Sensing and Spectral Phasor Analysis
Source: Anal Chem. 2023 Mar 16;95(12):5369–76. doi: 10.1021/acs.analchem.2c05708 (PMC10061367; doi:10.1021/acs.analchem.2c05708)

Supporting Information for

Determination of Intracellular Esterase Activity using Ratiometric Raman Sensing and Spectral Phasor Analysis

Henry J. Braddick,[†] William J. Tipping,[‡] Liam T. Wilson,[†] Harry S. Jaconelli,[‡] Emma K. Grant,[§] Karen Faulds,[‡]* Duncan Graham,[‡]* and Nicholas C. O. Tomkinson[†]*

[†]Department of Pure and Applied Chemistry, Thomas Graham Building, University of Strathclyde, 295 Cathedral Street, Glasgow, G1 1XL, U.K.

[‡]Centre for Molecular Nanometrology, Department of Pure and Applied Chemistry, Technology and Innovation Centre, University of Strathclyde, 99 George Street, Glasgow, G1 1RD, U.K.

[§]GlaxoSmithKline Medicines Research Centre, Gunnels Wood Road, Stevenage, SG1 2NY, U.K.

*Karen.Faulds@strath.ac.uk, *Duncan.Graham@strath.ac.uk, *Nicholas.Tomkinson@strath.ac.uk

Table of Contents

1. Experimental Procedures S3
1.1. Cell Culture S3
1.2. Raman Spectroscopy S6
1.3. SRS Microscopy S9
2. Supplementary Tables and Figures S12
3. Synthesis of Esterase Probes S16
3.1. General Information S16
3.2 Experimental Procedures S18
4. References S22
5. Copies of NMR Spectra S23

1. **Experimental Procedures**

1.1. Cell Culture

SK-BR-3 cells (ATCC® HTB-30™) were obtained from American Type Culture Collection (ATCC). U-87 MG (ATCC® HTB-14™) and HepG2 (ATCC® HB-8065™) cells were obtained from European Collection of Authenticated Cell Cultures (ECACC). HeLa cells (ATCC® CCL-2™) were gifted from the Strathclyde Institute of Pharmacy and Biomedical Sciences (Glasgow) as a subculture from a stock received from ECACC. SK-BR-3 cells were cultured in Rosewell Park Memorial Institute medium (RPMI 1640; GIBCO™, Fisher Scientific). HepG2 and HeLa cells were cultured in low glucose (1 g/L) Dulbecco's Modified Eagle Medium (DMEM). U-87 MG cells were cultured in Minimum Essential Medium (MEM). All media were supplemented with 10% foetal bovine serum (FBS, Gibco™, Fisher Scientific), 1% penicillin/streptomycin (Gibco™, 10000 U mL^{-1}, Fisher Scientific) and 1% amphotericin B (Gibco™, 250 mg mL^{-1}, Fisher Scientific). All cells were maintained at 37 °C and 5% CO_2 in a humidified incubator and were routinely sub-cultured at *ca*. 80% confluency. Compound treatments were performed using either PBS or media at the concentrations stated.

Spontaneous Raman Intracellular $\Delta\nu_{alkyne}$ Assessment. *Figure 3A*. HepG2 cells were plated on glass-bottomed culture dishes (35 mm high, Ibidi) at a concentration of 5×10^5 cells per well and incubated at 5% CO_2 and 37 °C for 24 h prior to compound treatment. For live cell imaging, cells were treated with compounds **5** or **4** (10 µM, diluted from a 20 mM stock solution in DMSO) in media and incubated at 5% CO_2 and 37 °C for 30 min. The dishes were then aspirated and washed with PBS (3 × 2 mL) before the cells were imaged in PBS. To simulate dead cells, cells were pre-treated with PFA (4% *v/v*) and Triton X-100 (0.05% *v/v*) in PBS for 2 h, before being washed with PBS (3 × 2 mL), treated with **5** or **4** (10 µM, diluted from a 20 mM stock solution in DMSO) in media, and incubated at 5% CO_2 and 37 °C for 30 min. The dishes were then aspirated and washed with PBS (3 × 2 mL) before imaging in PBS.

SRS Intracellular $\Delta\nu_{alkyne}$ Assessment. *Figure 3C*. HepG2 cells were plated in 6-well plates containing high precision glass coverslips (#1.5 H, 22×22 mm; Thorlabs) at a concentration of 5×10^5 cells per well and incubated in media at 5% CO_2 and 37 °C for 24 h prior to compound

treatment. For live cell imaging, cells were treated with **4** (10 µM, diluted from a 20 mM stock solution in DMSO) in media and incubated at 5% CO_2 and 37 °C for 30 min. The wells were then aspirated and washed with PBS (3 × 2 mL). Coverslips were then removed from the wells and affixed to microscope slides for imaging with a PBS boundary. To simulate dead cells, HepG2 cells were pre-treated with PFA (4% *v/v*) and Triton X-100 (0.05% *v/v*) in PBS for 2 h. The wells were then aspirated and washed with PBS (3 × 2 mL), treated with **4** (10 µM, diluted from a 20 mM stock solution in DMSO) in media, and incubated at 5% CO_2 and 37 °C for 30 min. The wells were then aspirated and washed with PBS (3 × 2 mL). Coverslips were then removed from the wells and affixed to microscope slides for imaging with a PBS boundary.

Colocalization Experiment. Figure 3D. HepG2 cells were plated in 6-well plates containing high precision glass coverslips (#1.5 H, 22×22 mm; Thorlabs) at a concentration of 5 × 10^5 cells per well and incubated in media at 5% CO_2 and 37 °C for 24 h prior to treatment. Cells were treated with a solution of **4** (10 µM, diluted from a 20 mM stock solution in DMSO) and the desired fluorescent stain (MitoTracker™ Red 250 nM; LysoTracker™ Green 62.5 nM; ER-Tracker™ Green 1 µM) in media and incubated at 5% CO_2 and 37 °C for 30 min. The wells were then aspirated and washed with PBS (3 × 2 mL). Coverslips were then removed from the wells and affixed to microscope slides for imaging with a PBS boundary.

Multimodal Cell Viability Stain Experiment. Figure 4A. HepG2 cells were plated in 6-well plates containing high precision glass coverslips (#1.5 H, 22×22 mm; Thorlabs) at a concentration of 5 × 10^5 cells per well and incubated in media at 5% CO_2 and 37 °C for 24 h prior to treatment. For live cell imaging, cells were treated with a solution of **4** (10 µM, diluted from a 20 mM stock solution in DMSO) and the cell viability stains (EthD-1, 4 µM; calcein AM, 2 µM) in media and incubated at 5% CO_2 and 37 °C for 30 min. The wells were then aspirated and washed with PBS (3 × 2 mL). Coverslips were then removed from the wells and affixed to microscope slides for imaging with a PBS boundary. To simulate dead cells, HepG2 cells were pre-treated with PFA (4% *v/v*) and Triton X-100 (0.05% *v/v*) in PBS for 2 h. The wells were then aspirated and washed with PBS (3 × 2 mL), treated with a solution of **4** (10 µM, diluted from a 20 mM stock solution in DMSO) and the cell viability stains (EthD-1, 4 µM; calcein AM, 2 µM) in media, and incubated at 5% CO_2 and 37 °C for 30 min. The wells were then aspirated and washed

with PBS (3 × 2 mL). Coverslips were then removed from the wells and affixed to microscope slides for imaging with a PBS boundary.

Localized UV Irradiation Experiment. Figure 5A. HepG2 cells were plated in high precision glass coverslips (#1.5 H, 75×25 mm; Thorlabs) with silicon perfusion chamber overlays (Grace Biolabs) at a concentration of 1 × 10^6 cells per mL and incubated in media at 5% CO_2 and 37 °C for 24 h. Prior to UV irradiation and imaging, the perfusion chamber was flushed with fresh media (80–200 µL). An SRS image was acquired using 1× zoom (512 × 512 frame). Using a 3× zoom, a selection of cells was targeted with UV irradiation (405 nm, ~5 mW) for 40 min. After which, a solution **4** (10 µM, diluted from a 20 mM stock solution in DMSO) in media (~200 µL) was perfused across the chamber. The cells were incubated for 10 min, following which, a SRS spectral sweep between 2253 and 2181 cm^{-1} (18 images) was acquired.

*Live/Fixed Phenol **5** Control Experiment. Figure S6A.* HepG2 cells were plated in 6-well plates containing high precision glass coverslips (#1.5 H, 22×22 mm; Thorlabs) at a concentration of 5 × 10^5 cells per well and incubated in media at 5% CO_2 and 37 °C for 24 h prior to compound treatment. For live cell imaging, cells were treated with **5** (10 µM, diluted from a 20 mM stock solution in DMSO) in media and incubated at 5% CO_2 and 37 °C for 30 min. The wells were then aspirated and washed with PBS (3 × 2 mL). The coverslips were then removed from the wells and affixed to microscope slides for imaging with a PBS boundary. To simulate dead cells, cells were pre-treated with PFA (4% *v/v*) and Triton X-100 (0.05% *v/v*) in PBS for 2 h. The wells were then aspirated and washed with PBS (3 × 2 mL), treated with **5** (10 µM, diluted from a 20 mM stock solution in DMSO) in media, and incubated at 5% CO_2 and 37 °C for 30 min. The wells were then aspirated and washed with PBS (3 × 2 mL). The coverslips were then removed from the wells and affixed to microscope slides for imaging with a PBS boundary.

HeLa/U-87 MG/SK-BR-3 Cell Line Experiments. Figure S7A. HeLa/U-87 MG/SK-BR-3 cells were plated in 6-well plates containing high precision glass coverslips (#1.5 H, 22×22 mm; Thorlabs) at a concentration of 5 × 10^5 cells per well and incubated in media at 5% CO_2 and 37 °C for 24 h prior to compound treatment. For live cell imaging, cells were treated with **4** (10 µM, diluted from a 20 mM stock solution in DMSO) in media and incubated at 5% CO_2 and 37 °C

for 30 min. The wells were then aspirated and washed with PBS (3 × 2 mL). Coverslips were then removed from the wells and affixed to microscope slides for imaging with a PBS boundary. To simulate dead cells, cells were pre-treated with PFA (4% *v/v*) and Triton X-100 (0.05% *v/v*) in PBS for 2 h. The wells were then aspirated and washed with PBS (3 × 2 mL), treated with **4** (10 µM, diluted from a 20 mM stock solution in DMSO) in media, and incubated at 5% CO_2 and 37 °C for 30 min. The wells were then aspirated and washed with PBS (3 × 2 mL). Coverslips were then removed from the wells and affixed to microscope slides for imaging with a PBS boundary.

High Wavenumber Spectral Phasor Experiment. Figure S8. HepG2 cells were plated in 6-well plates containing high precision glass coverslips (#1.5 H, 22×22 mm; Thorlabs) at a concentration of 5 × 10^5 cells per well and incubated in media at 5% CO_2 and 37 °C for 24 h prior to compound treatment. For live cell imaging, cells were treated with **4** (10 µM, diluted from a 20 mM stock solution in DMSO) in media and incubated at 5% CO_2 and 37 °C for 30 min. The wells were then aspirated and washed with PBS (3 × 2 mL). Coverslips were then removed from the wells and affixed to microscope slides for imaging with a PBS boundary. To simulate dead cells, cells were pre-treated with PFA (4% *v/v*) and Triton X-100 (0.05% *v/v*) in PBS for 2 h. The wells were then aspirated and washed with PBS (3 × 2 mL), treated with **4** (10 µM, diluted from a 20 mM stock solution in DMSO) in media, and incubated at 5% CO_2 and 37 °C for 30 min. The wells were then aspirated and washed with PBS (3 × 2 mL). Coverslips were then removed from the wells and affixed to microscope slides for imaging with a PBS boundary.

1.2. Raman Spectroscopy

Raman spectra were acquired on a Renishaw inVia Raman microscope equipped with a 532 nm Nd:YAG laser giving a maximum power of 50 mW using a 1800 lines/mm grating. Prior to spectral acquisition, the instrument was calibrated using the internal silicon standard at 520.5 cm^{-1} .

All spectra were processed using WiRE 4.4™. To process, spectral baselines were subtracted and spectra were smoothed using a Savitzky-Golay function with a polynomial order of 9 and a frame length of 3. Peak centres were determined using a non-linear Gauss. fitting function in Orgin2021. For cell maps, noise filtering was carried out prior to other processing.

Initial $\Delta\nu_{alkyne}$ in vitro Experiment. Figure 2C. Solutions of **4**, **5** and **6** (100 µM, diluted from 20 mM stock solutions in DMSO) in PBS were prepared in Eppendorf™ tubes and incubated at 37 °C for 1 h. The solutions were then transferred to a 96-well plate for imaging. Spectra of each solution were acquired using a 50×/NA 0.40 NPlanEPI objective lens, a laser power of 100% (36 mW), an acquisition time of 20 s and a single exposure. Three replicate measurements were made for each sample.

LoD Study with PLE. Figure 2D. PLE was purchased from Sigma-Aldrich (18 U/mg) and used without further purification. Solutions of **4**, **5** and **6** (100 µM, diluted from 20 mM stock solutions in DMSO) and varying concentrations of PLE (diluted from a 1 U/mL stock solution in PBS) in PBS:DMSO (8:2 *v/v*) were prepared in Eppendorf™ tubes and incubated at 37 °C for 1 h. The solutions were then transferred to a 96-well plate for imaging. Spectra of each solution were acquired using a 50×/NA 0.40 NPlanEPI objective lens, a laser power of 100% (36 mW), an acquisition time of 20 s and a single exposure. Three repeats were performed per condition.

Spontaneous Raman Intracellular $\Delta\nu_{alkyne}$ Assessment. *Figure 3A/3B*. Raman maps were acquired using λ_{ex} = 532 nm with a Nikon 60×, N.A. 1.0 NIR Apo water immersion objective, 5 µm step size in x and y, an acquisition time of 0.5 s, a laser power of 100% (36 mW) and a spectral centre of 2800 cm^{-1}. *Figure 3B*. Raman maps were acquired using λ_{ex} = 532 nm with a Nikon 60×/NA 1.0 NIR Apo water immersion objective, 5 µm step size in x and y, 0.5 s acquisition time, a laser power of 100% (36 mW) and a spectral centre of 2800 cm^{-1}. Three replicate maps were acquired from different culture plates for each condition. Average spectra were calculated for each cell map in MatLab R2022a, from which intensity ratios at 2212 and 2226 cm^{-1} were extracted.

PLE Heat Control Experiment. *Figure S1.* Solutions of **4** (100 μM, diluted from a 20 mM stock solution in DMSO) in PBS:DMSO (8:2 *v/v*), PBS:DMSO (8:2 *v/v*) containing PLE (1 U/mL) following heating at 90 °C for 3 h and PBS:DMSO (8:2 *v/v*) containing 0.5 U/mL PLE were prepared in Eppendorf™ tubes and incubated at 37 °C for 1 h. The solutions were then transferred to a 96-well plate for imaging. Spectra of each solution were acquired using a 50×/NA 0.40 NPlanEPI objective lens, a laser power of 100% (36 mW), an acquisition time of 20 s and a single exposure. Three repeats were performed per condition.

Timecourse Experiment. *Figure S2.* Solutions of **4**, **5** and **6** (100 μM, diluted from 20 mM stock solutions in DMSO) in PBS:DMSO (8:2 *v/v*) containing 0.08 U/mL PLE were prepared in Eppendorf™ tubes and incubated at 37 °C for the duration of the experiments. At 10 minute intervals, 7.5 μL of each solution was transferred to a CaF_2 microscope slide for imaging. Spectra were acquired using a 50×/NA 0.40 NPlanEPI objective lens, a laser power of 100% (36 mW), an acquisition time of 25 s and a single exposure. Three repeats were performed per condition.

Interference Agent Experiment. Figure S3A/S3B. Solutions of **4** and **6** (100 μM, diluted from 20 mM stock solutions in DMSO) and various interference agents (1 mM, diluted from 10 mM stock solutions in PBS) or PLE (0.1 U/mL, diluted from a 1 U/mL stock solution in PBS) in PBS:DMSO (8:2 *v/v*) were prepared in Eppendorf™ tubes and incubated at 37 °C for 1 h. The solutions were then transferred to a 96-well plate for imaging. Spectra of each solution were acquired using a 50×/NA 0.40 NPlanEPI objective lens, a laser power of 100% (36 mW), an acquisition time of 20 s and a single exposure. Three repeats were performed per condition.

pH Stability Experiment. Figure S4. Britton-Robinson buffers in 7:3 deionised water:EtOH were initially prepared and then pH adjusted by the addition of NaOH soln. (0.1 M) to the desired pH values of 5.31 and 9.43 as measured using a Thermo Scientific Orion Star™ pH meter. Individual wells of a 96-well plate were charged with a solution of **4** (500 μM, diluted from a 20 mM stock solution in DMSO) in each buffer and PBS (pH 7.4). Raman spectra of each solution were acquired every 10 min for 2 h using a 20×/NA 0.40 NPlanEPI objective lens, a laser power of 10% (~3.6 mW), an acquisition time of 5 s and a single exposure. A total of 3 repeats were performed per condition. At each pH value, spectra were unaffected in terms

of peak shift and intensity throughout the experiment, demonstrating the photostability of **4**. As a control experiment, the 96-well plate was also charged with a solution of **5** (500 µM, diluted from a 20 mM stock solution in DMSO) in each buffer and PBS (pH 7.4). Raman spectra of these solutions were acquired after 10 min using a 20×/NA 0.40 NPlanEPI objective lens, a laser power of 10% (~3.6 mW), an acquisition time of 5 s and a single exposure. Three repeats were performed per condition.

1.3. SRS Microscopy

An integrated laser system (picoEMERALD™ S, Applied Physics & Electronics, Inc.) was used to produce two synchronised laser beams at 80 MHz repetition rate. A fundamental Stokes beam (1031.4 nm, 2 ps pulse width) was intensity modulated by an electro-optic-modulator with >90% modulation depth, and a tuneable pump beam (700–960 nm, 2 ps pulse width, <1 nm (<10 cm^{-1}) spectral bandwidth) was produced by a built-in optical parametric oscillator. The pump and Stokes beams were spatially and temporally overlapped using two dichroic mirrors and a delay stage inside the laser system and coupled into an inverted laser-scanning microscope (Leica TCS SP8, Leica Microsystems) with optimised near-IR throughput. SRS images were acquired using 40× objective (HC PL IRAPO 40×, N.A. 1.10 water immersion lens) with a 9–48 µs pixel dwell time over a 512 × 512 frame. The Stokes beam was modulated with a 20 MHz EoM (Zurich Instruments). Forward scattered light was collected by a S1 N.A. 1.4 condenser lens (Leica Microsystems). Images were acquired at 12-bit image depth. The laser powers measured after the objective lens were in the range 10–30 mW for the pump beam only, 10–50 mW for the Stokes beam only and 20–70 mW (pump and Stokes beams). Wavenumber values were referenced against the CH_2 stretch of 1 micron polystyrene PMAA beads, which was determined using spontaneous Raman spectroscopy.

SRS Intracellular $\Delta\nu_{alkyne}$ Assessment. *Figure 3C*. HepG2 cells were prepared for imaging as described in the cell culture section. An SRS image at 2923 cm^{-1} (CH_3, protein) was captured before a spectral sweep between 2253 and 2181 cm^{-1} (18 images). Pseudo-Raman spectra were then generated from the spectral sweep data. All images were acquired at 512 × 512

pixels, 9–48 μs pixel dwell time. This process was then repeated twice more for each condition on different cells from the same population.

Colocalization Experiment. Figure 3D. HepG2 cells were prepared for imaging as described in the cell culture section. Fluorescence images were captured initially (MitoTracker™ Red λ_{ex} = 633 nm, λ_{em} = 640–750 nm; LysoTracker™ Green λ_{ex} = 488 nm, λ_{em} = 495–600 nm; ER-Tracker™ Green λ_{ex} = 488 nm, λ_{em} = 495–600 nm) before SRS images at 2923 cm^{-1} (CH_3, protein) and 2218 cm^{-1} (alkyne). All images were acquired at 512 × 512 pixels, 10 μs pixel dwell time. This process was then repeated twice more for each condition on different cells from within the same population. Merged images of **4** and organelle stains were generated in ImageJ and the Pearson's R values were calculated using the Coloc2 tool.

Multimodal Cell Viability Stain Experiment. Figure 4A. HepG2 cells were prepared for imaging as described in the cell culture section. Fluorescence images were captured initially (EthD-1 λ_{ex} = 514 nm, λ_{em} = 540–650 nm; calcein AM λ_{ex} = 488 nm, λ_{em} = 493–526 nm) before an SRS image at 2923 cm^{-1} (CH_3, protein) and a SRS spectral sweep (2253-2181 cm^{-1}, 18 images). Images at 2232 cm^{-1} and 2219 cm^{-1} were taken from the corresponding images of the SRS spectral sweeps. All images were acquired at 512 × 512 pixels, 9–48 μs pixel dwell time. This process was then repeated twice more for each condition on different cells from within the same population.

Localized UV Irradiation Experiment. Figure 5A. HepG2 cells were prepared for imaging in a perfusion chamber as described in the cell culture section. An SRS image at 2923 cm^{-1} (CH_3, protein) at 1× zoom was captured prior to UV irradiation of the cells. The field-of-view was then adjusted to 3× zoom to encompass a single cluster of cells, to which UV irradiation (100% laser power, 5 mW) was applied for 40 min. Cells were then treated with **4** (10 μM, diluted from a 20 mM stock solution in DMSO) in PBS and imaged again. An SRS image at 2923 cm^{-1} (CH_3, protein) of the UV irradiated cluster of cells was captured before the field of view was expanded to its original level. An SRS image at 2923 cm^{-1} (CH_3, protein) and a SRS spectral sweep (2253-2181 cm^{-1}, 18 images) were then captured. Images at 2232 cm^{-1} and 2219 cm^{-1} were taken from the corresponding images of the SRS spectral sweeps. All images were acquired at 512 × 512 pixels, 9–48 μs pixel dwell time.

*Live/Fixed Phenol **5** Control Experiment. Figure S6A.* HepG2 cells were prepared for imaging as described in the cell culture section. An SRS image at 2923 cm^{-1} (CH_3, protein) and a SRS spectral sweep (2253-2181 cm^{-1}, 18 images) were then captured. Images at 2232 cm^{-1} and 2219 cm^{-1} were taken from the corresponding images of the SRS spectral sweeps. All images were acquired at 512 × 512 pixels, 9–48 µs pixel dwell time. This process was then repeated twice more for each condition on different cells from within the same population.

HeLa/U-87 MG/SK-BR-3 Cell Line Experiments. Figure S7A. HeLa, U-87 MG and SK-BR-3 cells were prepared for imaging as described in the cell culture section. An SRS image at 2923 cm^{-1} (CH_3, protein) and a SRS spectral sweep (2253–2181 cm^{-1}, 18 images) were then captured. Images at 2232 cm^{-1} and 2219 cm^{-1} were taken from the corresponding images of the SRS spectral sweeps. All images were acquired at 512 × 512 pixels, 9–48 µs pixel dwell time. This process was then repeated twice more for each condition on different cells from within the same population.

High Wavenumber Spectral Phasor Experiment. Figure S8. HepG2 cells were prepared for imaging as described in the cell culture section and a SRS spectral sweep (3050–2803 cm^{-1}, 40 images) was then captured. All images were acquired at 512 × 512 pixels, 9–48 µs pixel dwell time.

2. Supplementary Tables and Figures

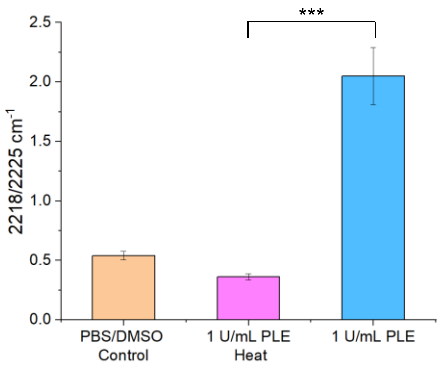

*Figure S1. PLE heat control experiment. AM ester **4** (100 µM) was dissolved in PBS:DMSO (8:2 v/v, orange), PBS:DMSO (8:2 v/v) containing 1 U/mL PLE following heating at 90 °C for 3 h (pink) and PBS:DMSO (8:2 v/v) containing 1 U/mL PLE (blue). (532 nm, 1 × 20 s exposure, 50× lens. Spectra were acquired after 1 h incubation at 37 °C). *** T test $p \leq 1 \times 10^{-3}$.*

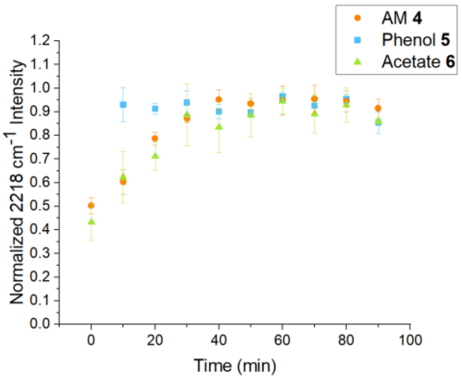

*Figure S2. Timecourse assessment of the reactivity of **4** and **6** toward PLE. Compounds **4**, **5** and **6** (100 µM) were dissolved in PBS:DMSO (8:2 v/v) containing 0.08 U/mL PLE and incubated at 37 °C for the duration of the experiments. Spectra were acquired at 10 minute intervals (532 nm, 1 × 25 s exposure, 50× lens). For t = 0 measurements, **4** and **6** (100 µM) were dissolved in PBS:DMSO (8:2 v/v) and imaged (532 nm, 1 × 25 s exposure, 50× lens). The intensities at 2218 cm^{-1} were then extracted and normalized to 1.*

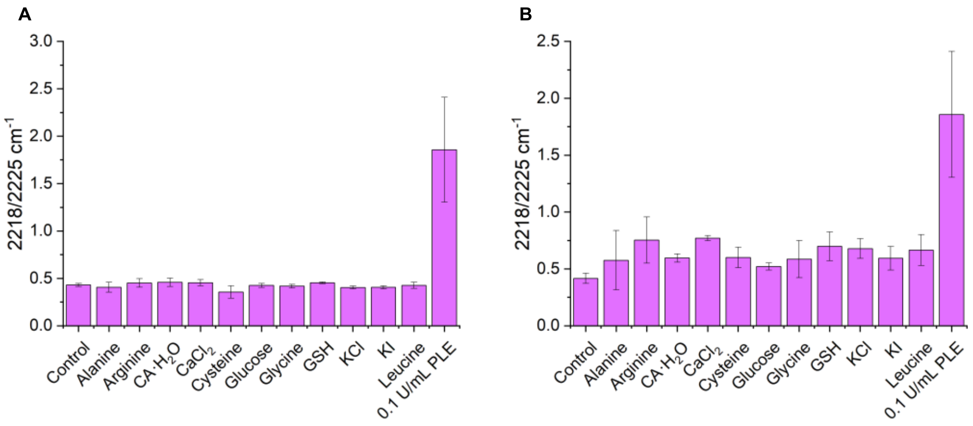

*Figure S3. Selectivity assessment of **4** and **6**. **A:** Stability test of AM ester **4** in the presence of a number of interference agents. (100 µM **4**, PBS:DMSO (8:2 v/v), 1 mM interference agent or 0.1 U/mL PLE, 532 nm, 1 × 20 s exposure, 50× lens. Spectra were*

*acquired after 1 h incubation at 37 °C. Control = 100 µM **4** in PBS:DMSO (8:2 v/v); CA·H_2O = citric acid monohydrate). **B**: Stability test of acetate **6** in the presence of a number of interference agents. (100 µM **6**, PBS:DMSO (8:2 v/v), 1 mM interference agent or 0.1 U/mL PLE, 532 nm, 1 × 20 s exposure, 50× lens. Spectra were acquired after 1 h incubation at 37 °C. Control = 100 µM **6** in PBS:DMSO (8:2 v/v); CA·H_2O = citric acid monohydrate).*

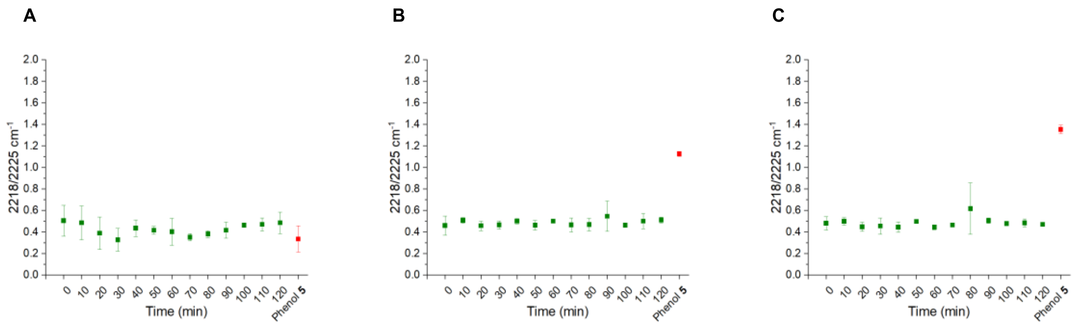

*Figure S4. pH stability assay of **4** (500 µM) in pH 5.31 buffer (**A**), PBS (pH 7.4, **B**), and pH 9.43 buffer (**C**). (532 nm, 1 × 5 s exposure, 20× lens). Britton-Robinson buffers in 7:3 deionised water:EtOH were initially prepared and then pH adjusted by the addition of NaOH soln. (0.1 M) to the desired pH values of 5.31 and 9.43. Phenol **5** (500 µM) in each buffer/PBS was used as a control experiment and analysed using the same imaging parameters.*

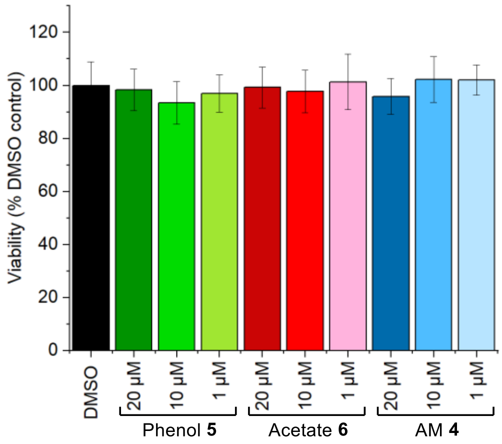

*Figure S5. Cytotoxicity study of phenol **5**, acetate **6** and AM ester **4**. HepG2 cells were treated with varying concentrations of **4**, **5** or **6** in PBS, or a DMSO control for 8 h (n = 2 plates per condition). The resultant viable cell-count in each plate was then quantified using the Alamar blue assay.*

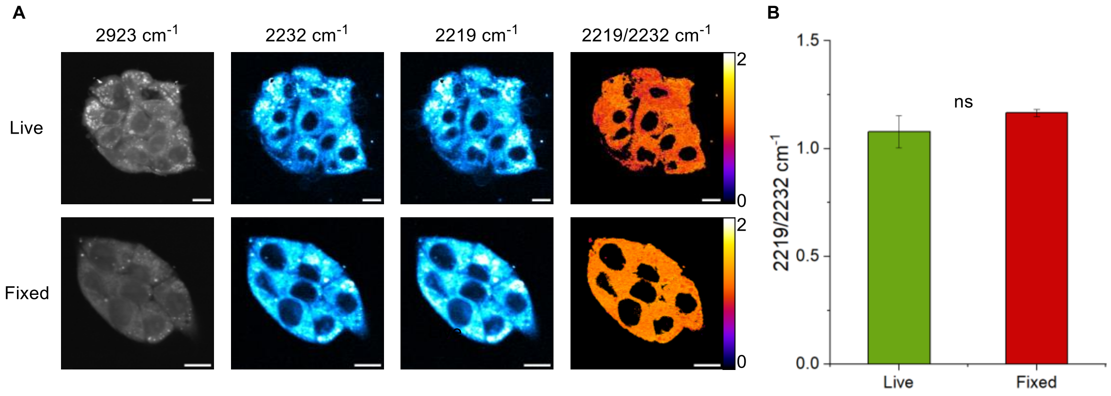

*Figure S6. Ratiometric analysis of difluorophenol **5** in live and fixed HepG2 cells. **A**: **5** in live and fixed HepG2 cells. (To fix, cells were pre-treated with PFA (4% v/v) and Triton X-100 (0.05% v/v) in PBS for 2 h prior to addition of **5**). Images were acquired after treatment with **5** (10 µM) in media for 30 min. All images were acquired at 512 × 512 pixels, 9–48 µs pixel dwell time. Images at 2232 cm^{-1} and 2219 cm^{-1} were taken from the corresponding images of SRS spectral sweeps (2253–2181 cm^{-1}, 18 images). False colors and scale bars representing 10 µm were applied in ImageJ. Ratio bars show the Fire LUT scaled between values of 0 and 2. **B**: Ratio of the intensities at 2219 cm^{-1} and 2232 cm^{-1} in live and fixed HepG2 cells. Pseudo-Raman spectra were generated from >3 cells in each spectral sweep (2253–2181 cm^{-1}, 18 images) and the intensities at 2219 cm^{-1} and 2232 cm^{-1} were extracted. ns T test $p > 0.05$.*

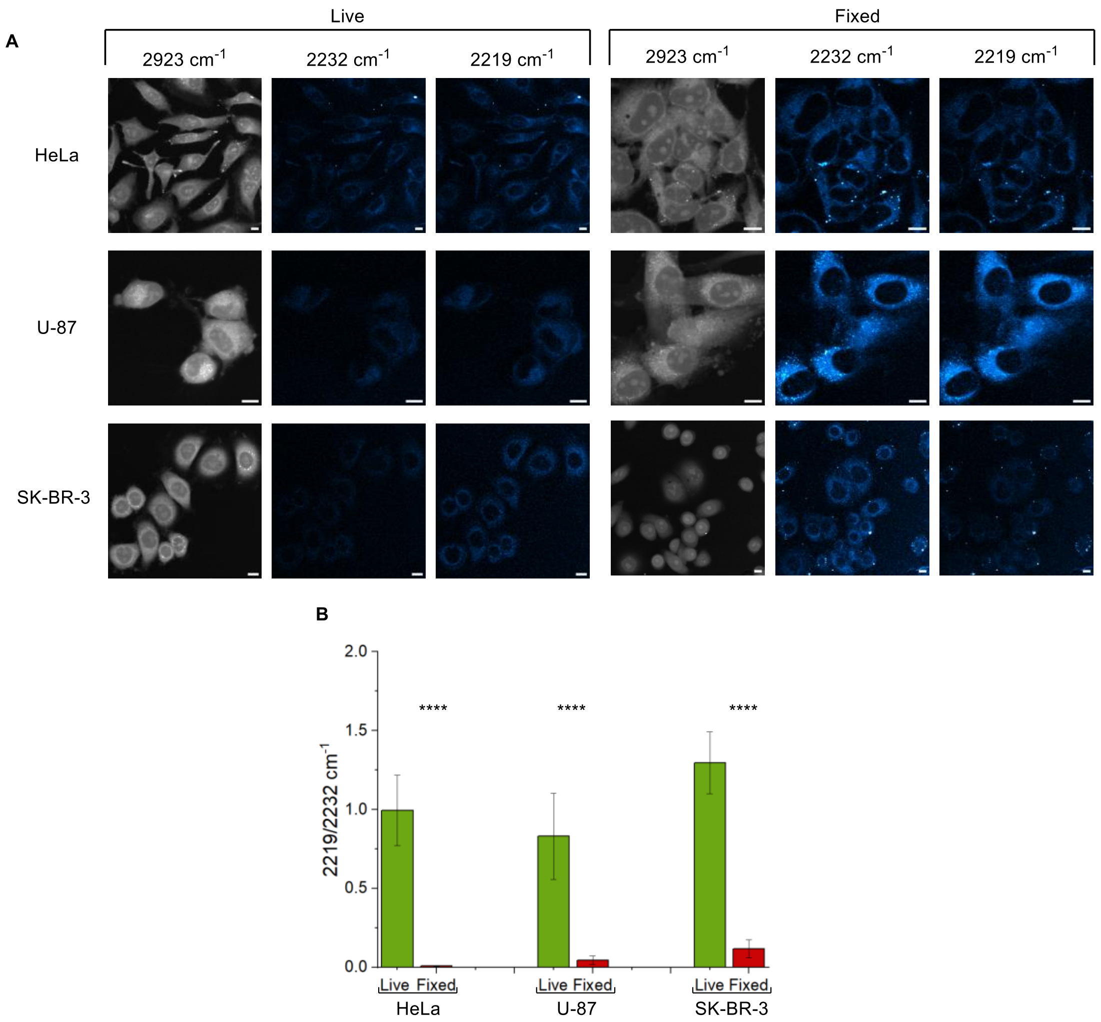

*Figure S7. Ratiometric analyses of **4** as an intracellular esterase sensor in different cell lines. **A**: Ratiometric study of **4** in live and fixed HeLa, U-87 and SK-BR-3 cells. (To fix, cells were pre-treated with PFA (4% v/v) and Triton X-100 (0.05% v/v) in PBS for 2 h prior to treatment with **4**). Images were acquired after treatment with **4** (10 µM) in media for 30 min. All images were acquired at 512 × 512 pixels, 9–48 µs pixel dwell time. Images at 2232 cm^{-1} and 2219 cm^{-1} were taken from the corresponding images of SRS spectral sweeps (2253–2181 cm^{-1}, 18 images). False colors and scale bars representing 10 µm were applied in ImageJ. **B**: Ratio of the intensities at 2219 cm^{-1} and 2232 cm^{-1} in live and fixed HeLa, U-87 and SK-BR-3 cells. Pseudo-Raman spectra were generated from >3 cells in each spectral sweep (2253–2181 cm^{-1}, 18 images) and the intensities at 2219 cm^{-1} and 2232 cm^{-1} were extracted. **** T test $p \leq 1 \times 10^{-4}$.*

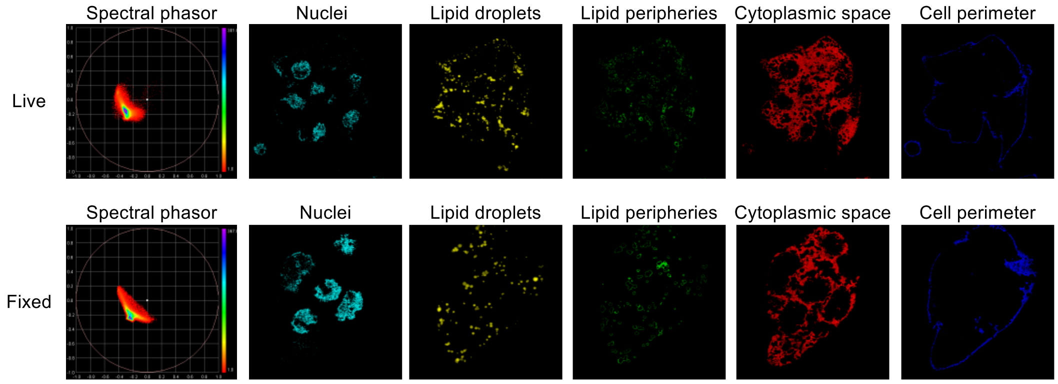

*Figure S8. Spectral phasor analysis of high wavenumber SRS spectral sweeps (3050–2803 cm^{-1}, 40 images) of live and fixed HepG2 cells treated with **4**. (To fix, cells were pre-treated with PFA (4% v/v) and Triton X-100 (0.05% v/v) in PBS for 2 h prior*

*to treatment with **4**). Images were acquired after treatment with **4** (10 μM) in media for 30 min. All images were acquired at 512 × 512 pixels, 9–48 μs pixel dwell time. SRS spectral sweeps were background subtracted on ImageJ and phasor plots were generated using an ImageJ plugin. The corresponding images of cellular compartments were then generated from appropriate ROIs on the spectral phasor plot.*

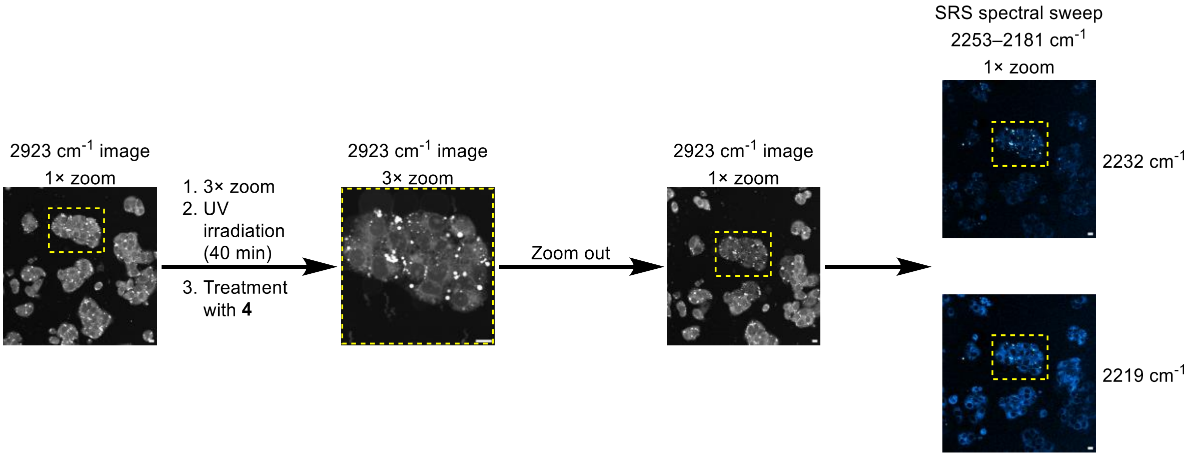

Figure S9. Experimental workflow of the localized UV irradiation experiment presented in Figure 5A.

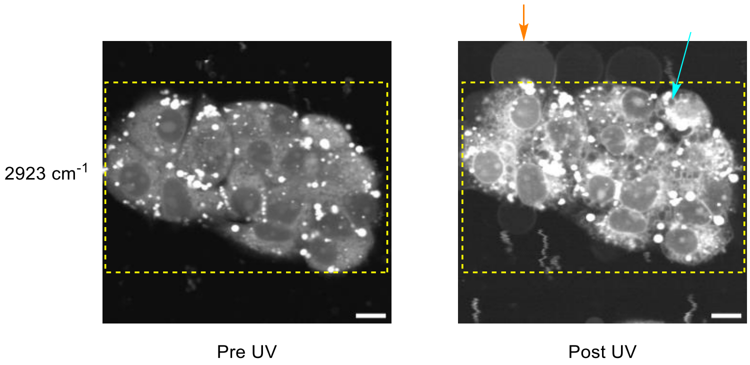

Figure S10. Expanded image from Figure 5A displaying cellular blebbing (orange arrow) and vacuole formation (blue arrow) following localized UV irradiation. (Brightness and contrast settings in ImageJ have been adjusted for the post UV image to better display these features).

3. Synthesis of Esterase Probes

3.1. General Information

All reagents were obtained from Sigma-Aldrich, Alfa Aesar or Fluorochem and used without purification. Anhydrous solvents tetrahydrofuran (THF), dichloromethane (CH_2Cl_2), diethyl ether (Et_2O), hexane and toluene were obtained from a PureSolv MD 5 Solvent Purification System by Innovative Technology Inc., and handled under inert atmosphere without further

purification. Solvents were acquired from commercial sources and used without further purification unless otherwise stated. Flash column chromatography was carried out using Fischer Scientific chromatography grade silica 60 Å particle size 35–70 micron. Analytical thin layer chromatography was carried out using aluminium-backed plates coated with Machery-Nagel pre-coated TLC sheets, coated in 0.20 mm silica gel 60 with UV_{254} fluorescent indicator. Sheets were visualized under UV light (at 254 nm) or stained using potassium permanganate solution. Nuclear magnetic resonance (NMR) spectra were recorded on a Bruker Avance III spectrometer operating at 400 MHz (^{1}H) and 101 MHz (^{13}C). Chemical shifts were reported in parts per million (ppm) in the scale relative to $CDCl_3$, 7.26 ppm for ^{1}H NMR and 77.16 for ^{13}C NMR; $(CD_3)_2SO$ (dimethylsulfoxide), 2.50 ppm for ^{1}H NMR and 39.52 for ^{13}C NMR. Multiplicities are abbreviated as: s, singlet; d, doublet; t, triplet; q, quartet; dd, doublet of doublets; ddd, doublet of doublets of doublets; td, triplet of doublets; app.t, apparent triplet; app.td, apparent triplet of doublets; app.p, apparent pentet; hept, heptet; dhept, doublet of heptets; m, multiplet; br, broad. Coupling constants are measured in Hertz (Hz). Low-resolution mass spectra (LRMS) were recorded on an Agilent 6130 single quadrupole with APCI/ESI dual source, on a ThermoQuest Finnigan LCQ DUO electrospray, or on an Agilent 7890A GC system equipped with a 30 m DB5MS column connected to a 5975C inert XL CI MSD with TripleAxis Detector and were determined using atmospheric pressure chemical ionization (APCI) unless otherwise stated. ESI refers to electrospray ionization, CI refers to chemical ionization (methane) and EI refers to electron ionization. Melting points were obtained on a Gallenkamp Griffin MPA350.BM2.5 device. Infrared spectra were recorded on an Agilent Technologies 5500 series FTIR. *In vacuo* refers to evaporation under reduced pressure using a rotary evaporator connected to a diaphragm pump, followed by the removal of trace volatiles using a high vacuum (oil) pump.

3.2 Experimental Procedures

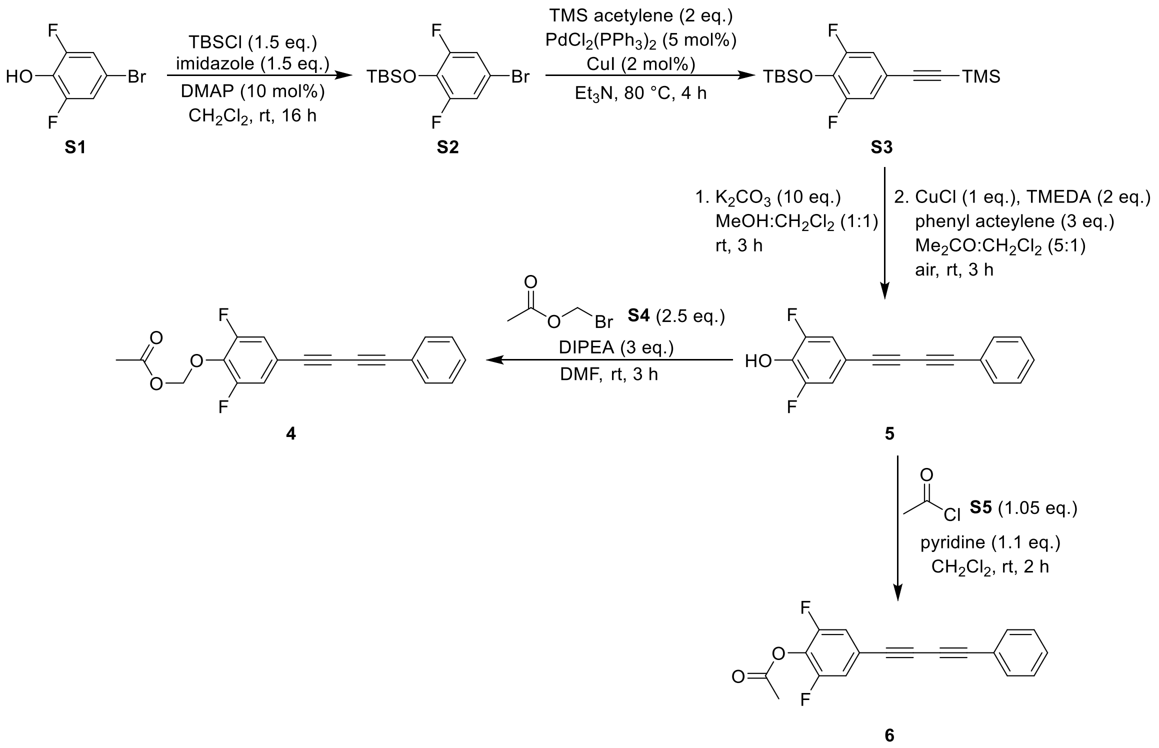

*Figure S11. Synthesis of AM ester **4** and acetate **6**.*

(4-Bromo-2,6-difluorophenoxy)(*tert*-butyl)dimethylsilane **S2**.[1]

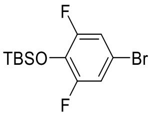

A flame dried microwave vial was charged with 4-bromo-2,6-difluorophenol **S1** (1.05 g, 5.00 mmol), 4-dimethylaminopyridine (DMAP, 61 mg, 0.50 mmol), imidazole (510 mg, 7.50 mmol) and TBSCl (1.13 g, 7.50 mmol). The microwave vial was then purged with N_2 gas prior to the addition of anhydrous CH_2Cl_2 (12.5 mL). The reaction was stirred at room temperature for 16 h before being partitioned with sat. NH_4Cl solution (20 mL). The aqueous layer was extracted with CH_2Cl_2 (3 × 15 mL) and the combined organics were washed with water (20 mL) and brine (20 mL). The organic phase was dried over $MgSO_4$ and concentrated *in vacuo*. Purification by flash chromatography on silica gel (100% petroleum ether 60–80) afforded the *title compound* as a colorless oil (1.53 g, 4.73 mmol, 95%). **FTIR (ATR, cm^{-1})**: 2934, 2863, 1582, 1506, 1428, 1314, 1255; **^{1}H NMR (400 MHz, $CDCl_3$)**: δ 7.08–7.01 (m, 2H), 1.00 (s, 9H), 0.19 (app. t, J = 1.1 Hz, 6H); **^{19}F NMR (376 MHz, $CDCl_3$, ^{1}H decoupled)**: δ -126.8; **^{13}C NMR (101 MHz,

$CDCl_3$): δ 155.3 (dd, J_{CF} = 249.4, 6.4 Hz), 132.4, 115.8 (dd, J_{CF} = 18.4, 8.5 Hz), 111.4 (t, J_{CF} = 11.3 Hz), 25.6 (s), 18.6 (s), -4.8 (s); **LRMS (ES + APCI)**: *m/z* calc. 324.0 found 206.9 $[M\text{-}TBSH]^-$.

tert-Butyl(2,6-difluoro-4-((trimethylsilyl)ethynyl)phenoxy)dimethylsilane **S3**.[1]

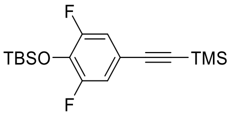

A flame dried microwave vial was charged with aryl bromide **S2** (972 mg, 3.00 mmol), $PdCl_2(PPh_3)_2$ (105 mg, 0.15 mmol) and CuI (11.4 mg, 0.06 mmol) before being sealed and purged with N_2 gas. Triethylamine (Et_3N, 12.0 mL) was added and the mixture was degassed with 4 freeze-pump-thaw cycles. The mixture was then warmed to ambient temperature prior to the addition of TMS acetylene (828 µL, 6.00 mmol), after which the reaction was stirred at 80 °C for 4 h. The mixture was allowed to cool to ambient temperature, diluted with Et_2O (50 mL) and filtered through Celite®. The filtrate was concentrated *in vacuo* and purification by flash chromatography on silica gel (100% hexane) afforded the *title compound* as a colorless oil (792 mg, 2.33 mmol, 78%). **FTIR (ATR, cm^{-1})**: 2934, 2863, 2165, 1519, 1357, 1253; **^1H NMR (400 MHz, $CDCl_3$)**: δ 7.02–6.94 (m, 2H), 1.00 (s, 9H), 0.23 (s, 9H), 0.19 (app. t, J = 1.09 Hz, 6H); **^{19}F NMR (376 MHz, $CDCl_3$, ^1H decoupled)**: δ -128.7; **^{13}C NMR (101 MHz, $CDCl_3$)**: δ 154.7 (dd, J_{CF} = 245.4, 6.2 Hz), 133.9 (s), 116.0–115.4 (m), 103.0 (t, J_{CF} = 3.6 Hz), 94.8 (s), 25.6 (s), 18.6 (s), 0.0 (s), -4.8 (s); **LRMS (ES + APCI)**: *m/z* calc. 340.2 found 225.1 $[M\text{-}TBSH]^-$.

2,6-Difluoro-4-(phenylbuta-1,3-diyn-1-yl)phenol **5**.[1]

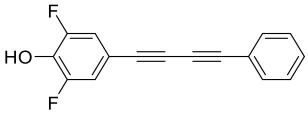

A microwave vial was charged with TMS protected alkyne **S3** (625 mg, 1.83 mmol), K_2CO_3 (2.53 g, 18.3 mmol) and a mixture of MeOH/CH_2Cl_2 (1:1, 14 mL). The mixture was stirred at rt for 2 h before being diluted with water, neutralised by the addition of 1 M HCl and extracted with CH_2Cl_2 (3 × 10 mL). The combined organics were washed with brine (15 mL) and dried over $MgSO_4$ before being concentrated *in vacuo*. The crude terminal alkyne was telescoped into the next step without further purification. A suspension of CuCl (181 mg, 1.83 mmol) and *N,N,N*',*N*'-tetramethylethylenediamine (TMEDA, 548 µL, 3.66 mmol) in acetone (18.3 mL) was prepared and bubbled with air for 15 min. A solution of the terminal alkyne and

phenylacetylene (602 µL, 5.49 mmol) in CH_2Cl_2 (3.66 mL) was then prepared and added to the CuCl/TMEDA suspension. The reaction was stirred at rt for 3 h whilst open to air before being partitioned with sat. NH_4Cl solution (30 mL). The aqueous phase was extracted with EtOAc (3 × 10 mL) and the combined organics were washed with brine (15 mL) and dried over $MgSO_4$ before being concentrated *in vacuo*. Purification by flash chromatography on silica gel (100% CH_2Cl_2) afforded the *title compound* as an off white solid (217 mg, 0.85 mmol, 46%). **FTIR (ATR, cm^{-1})**: 3539, 2863, 1582, 1506, 1428, 1314, 1255; **^{1}H NMR (400 MHz, $CDCl_3$)**: δ 7.55–7.50 (m, 2H), 7.42–7.32 (m, 3H), 7.14–7.05 (m, 2H), 5.32 (s, 1H); **^{19}F NMR (376 MHz, $CDCl_3$, ^{1}H decoupled)**: δ -134.6; **^{13}C NMR (101 MHz, $CDCl_3$)**: δ 151.3 (dd, J_{CF} = 244.5, 6.5 Hz), 134.8 (t, J_{CF} = 16.0 Hz), 132.7 (s), 129.6 (s), 128.6 (s), 121.6 (s), 116.4–116.1 (m), 113.4 (t, J_{CF} = 10.6 Hz), 82.2 (s), 79.2 (t, J_{CF} = 3.7 Hz), 74.3 (s), 73.6 (s); **LRMS (ES + APCI)**: m/z calc. 254.1 found 253.0 $[M-H]^-$.

(2,6-Difluoro-4-(phenylbuta-1,3-diyn-1-yl)phenoxy)methyl acetate **4**.

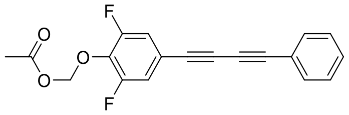

A solution of difluorophenol **5** (26 mg, 0.10 mmol) in dimethylformamide (DMF, 0.75 mL) was prepared before bromomethyl acetate (**S4**, 28 µL, 0.25 mmol) and diisopropylethylamine (DIPEA, 35 µL, 0.30 mmol) were added sequentially. The reaction was stirred at rt for 4 h before being quenched with brine (2 mL). The aqueous phase was extracted with EtOAc (3 × 3 mL) and the combined organics were washed with brine (3 × 1 mL), dried over $MgSO_4$ and concentrated *in vacuo*. Purification by flash column chromatography (5% EtOAc/petroleum ether 60–80) afforded the *title compound* as an off white solid (12 mg, 0.04 mmol, 37%). **M.P.:** 78–82 °C; **FTIR (ATR, cm^{-1})**: 3064, 2925, 2226, 1761, 1573, 1515; **^{1}H NMR (400 MHz, $CDCl_3$)**: δ 7.56–7.51 (m, 2H), 7.43–7.32 (m, 3H), 7.13–7.06 (m, 2H), 5.67 (s), 2.12 (s); **^{19}F NMR (376 MHz, $CDCl_3$, ^{1}H decoupled)**: δ -127.0; **^{13}C NMR (101 MHz, $CDCl_3$)**: δ 169.9 (s), 155.8 (dd, J_{CF} = 250.2, 6.1 Hz), 132.8 (s), 129.7 (s), 128.7 (s), 121.4 (s), 118.8 (t, J_{CF} = 11.0 Hz), 116.8–116.5 (m), 88.4 (t, J_{CF} = 3.0 Hz), 82.9 (s), 78.6 (t, J = 3.4 Hz), 75.6 (s), 73.4 (s), 20.8 (s). **LRMS (ES + APCI)**: m/z calc. 326.1 found 327.0 $[M+H]^+$. **HRMS (ESI)**: $[M+Na]^+$ calc for $C_{19}H_{12}O_3F_2Na$ 349.06467 found 349.0641.

2,6-Difluoro-4-(phenylbuta-1,3-diyn-1-yl)phenyl acetate **6.**

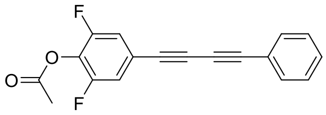

A solution of difluorophenol **5** (26 mg, 0.10 mmol) and pyridine (9 µL, 0.14 mmol) in anhydrous CH_2Cl_2 (0.4 mL) was prepared and acyl chloride (**S5**, 8 µL, 0.11 mmol) was added dropwise. The reaction was stirred at rt for 2 h before being diluted with 1 M HCl (1 mL). The aqueous phase was extracted with CH_2Cl_2 (3 × 3 mL) before the combined organics were washed with brine (3 mL), dried over $MgSO_4$, and concentrated *in vacuo*. Purification by flash column chromatography (5% EtOAc/petroleum ether 60–80) afforded the *title compound* as an off white solid (18 mg, 0.06 mmol, 61%). **M.P.:** 125–128 °C; **FTIR (ATR, cm^{-1})**: 2921, 2852, 2226, 1774, 1595, 1513; **^{1}H NMR (400 MHz, CDCl$_3$)**: δ 7.56–7.51 (m, 2H), 7.43–7.32 (m, 3H), 7.16–7.10 (m, 2H), 2.38 (s, 3H); **^{19}F NMR (376 MHz, CDCl$_3$, ^{1}H decoupled)**: δ -124.9; **^{13}C NMR (101 MHz, CDCl$_3$)**: δ 167.2 (s), 155.0 (dd, J_{CF} = 251.4, 4.8 Hz), 132.8 (s), 129.8 (s), 128.7 (s), 121.4 (s), 120.7 (t, J_{CF} = 10.8 Hz), 116.6–116.3 (m), 83.1 (s), 78.5 (t, J_{CF} = 3.7 Hz), 76.0 (s), 73.3 (s), 20.2 (s); **LRMS (ES + APCI)**: m/z calc. 296.1 found 297.0 $[M+H]^+$. **HRMS (ESI)**: $[M+Na]^+$ calc for $C_{18}H_{10}O_2F_2Na$ 319.05411 found 319.0534.

4. References

1 Wilson, L. T.; Tipping, W. J.; Jamieson, L. E.; Wetherill, C.; Henley, Z.; Faulds, K.; Graham, D.; MacKay, S. P.; Tomkinson, N. C. O. A new class of ratiometric small molecule intracellular pH sensors for Raman microscopy. *Analyst* **2020**, 145, 15, 5289–5298. DOI: 10.1039/D0AN00865F

5. Copies of NMR Spectra

^{1}H NMR spectrum of **S2** (400 MHz, $CDCl_3$)

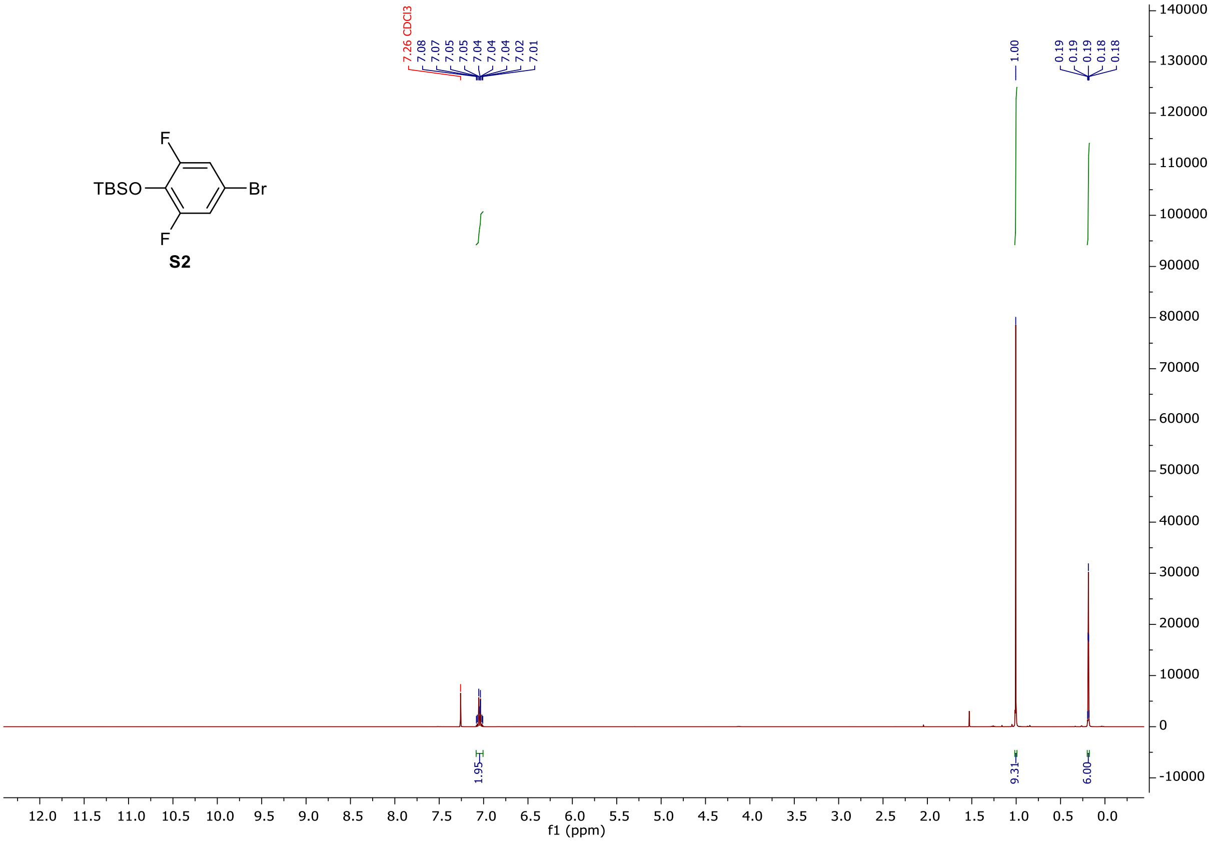

^{19}F NMR spectrum of **S2** (376 MHz, $CDCl_3$)

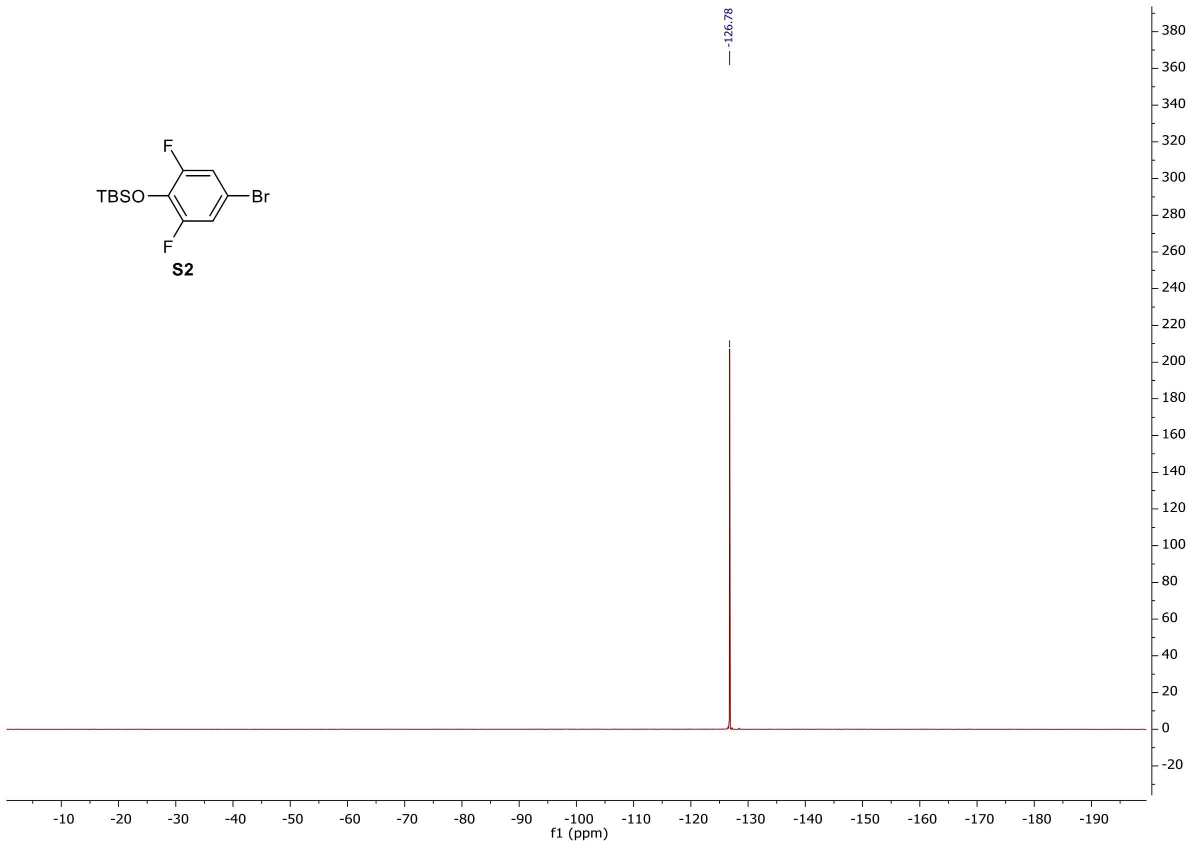

^{13}C NMR spectrum of **S2** (101 MHz, $CDCl_3$)

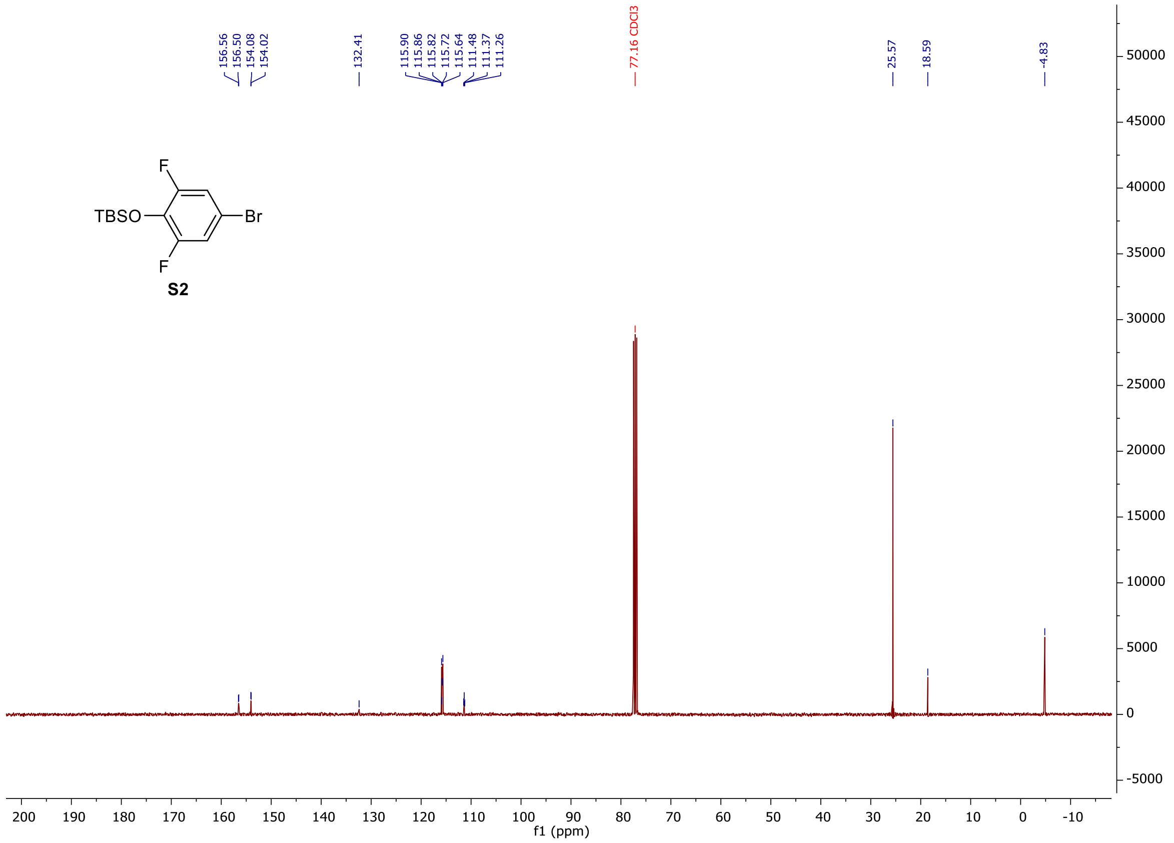

^{1}H NMR spectrum of **S3** (400 MHz, $CDCl_3$)

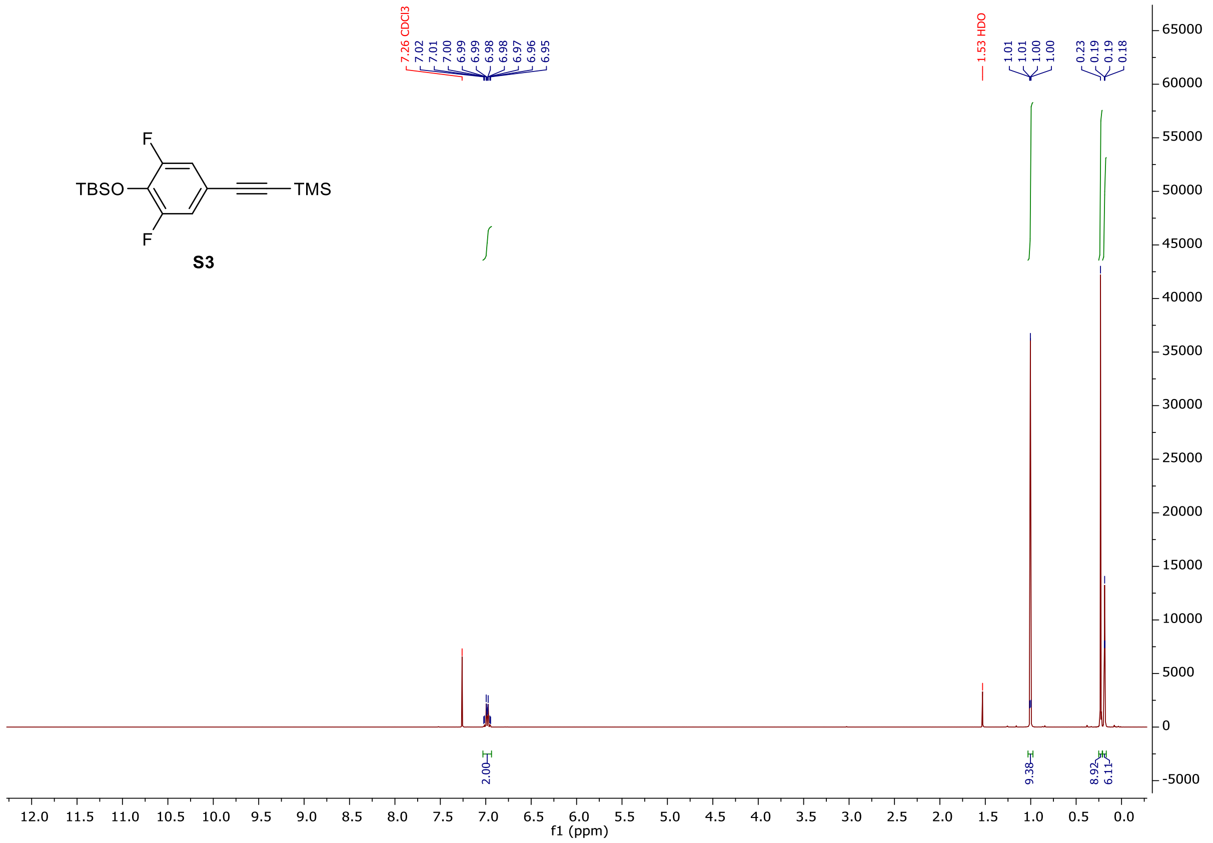

^{19}F NMR spectrum of **S3** (376 MHz, $CDCl_3$)

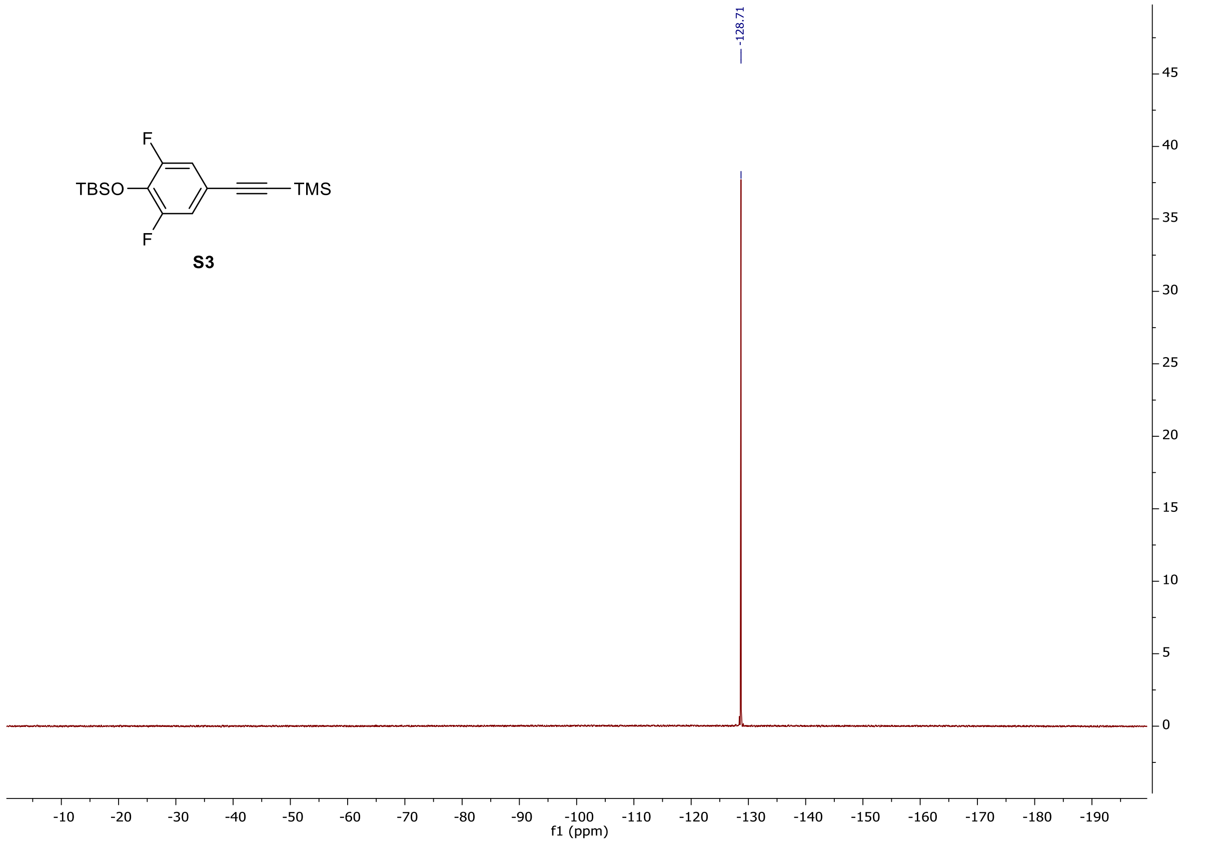

^{13}C NMR spectrum of **S3** (101 MHz, $CDCl_3$)

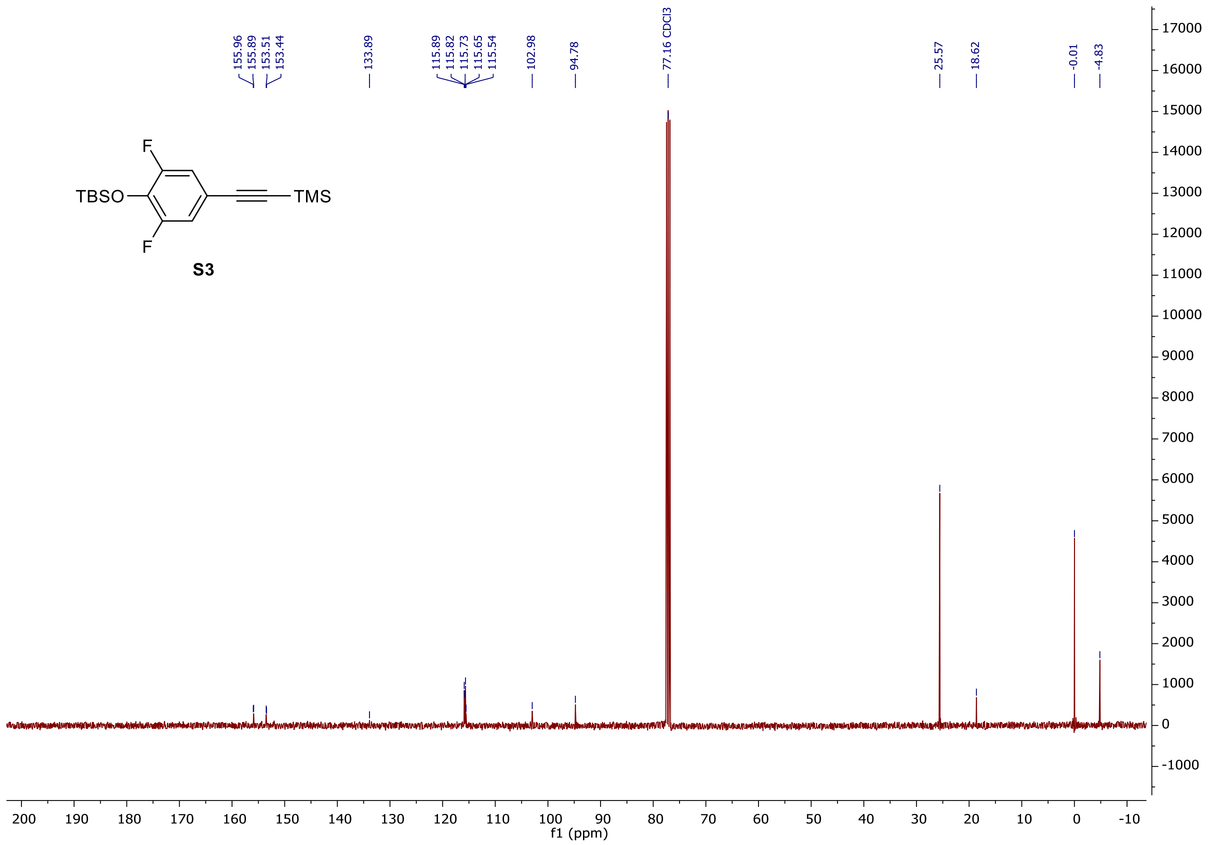

^{1}H NMR spectrum of **5** (400 MHz, $CDCl_3$)

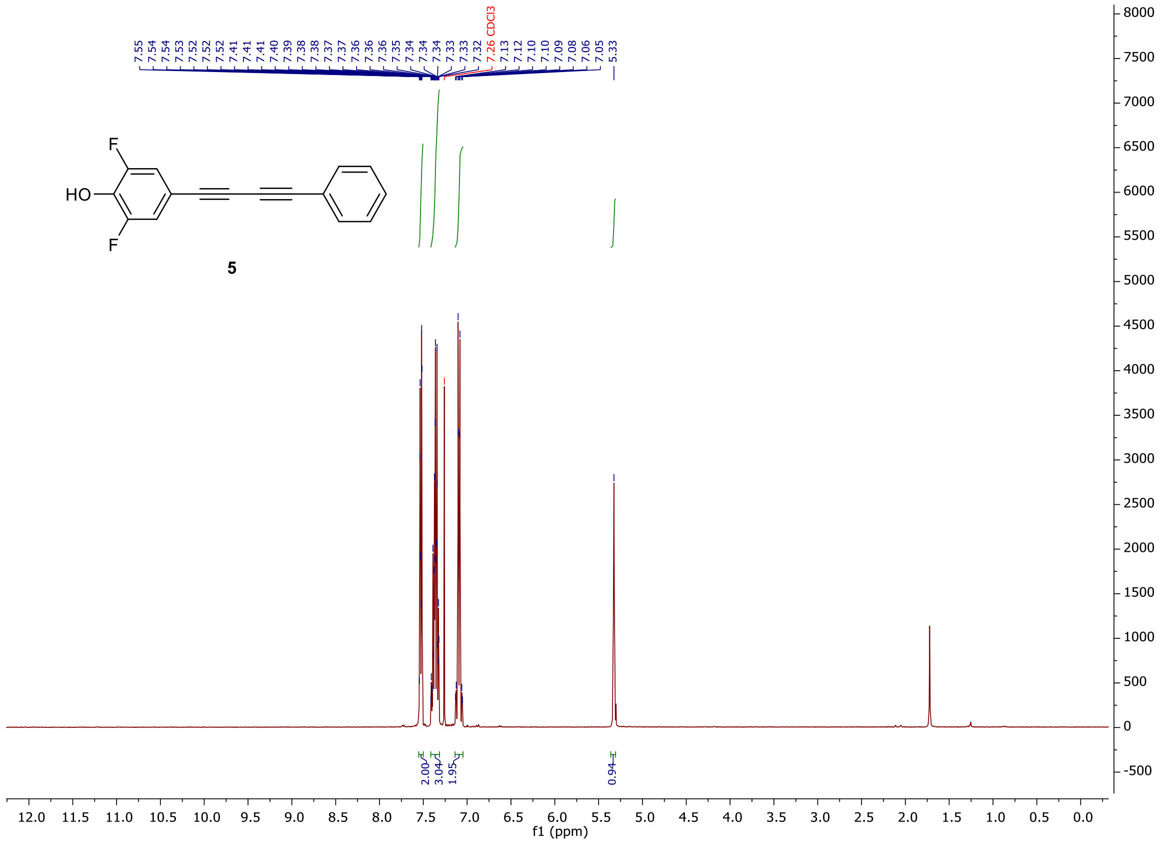

^{19}F NMR spectrum of **5** (376 MHz, $CDCl_3$)

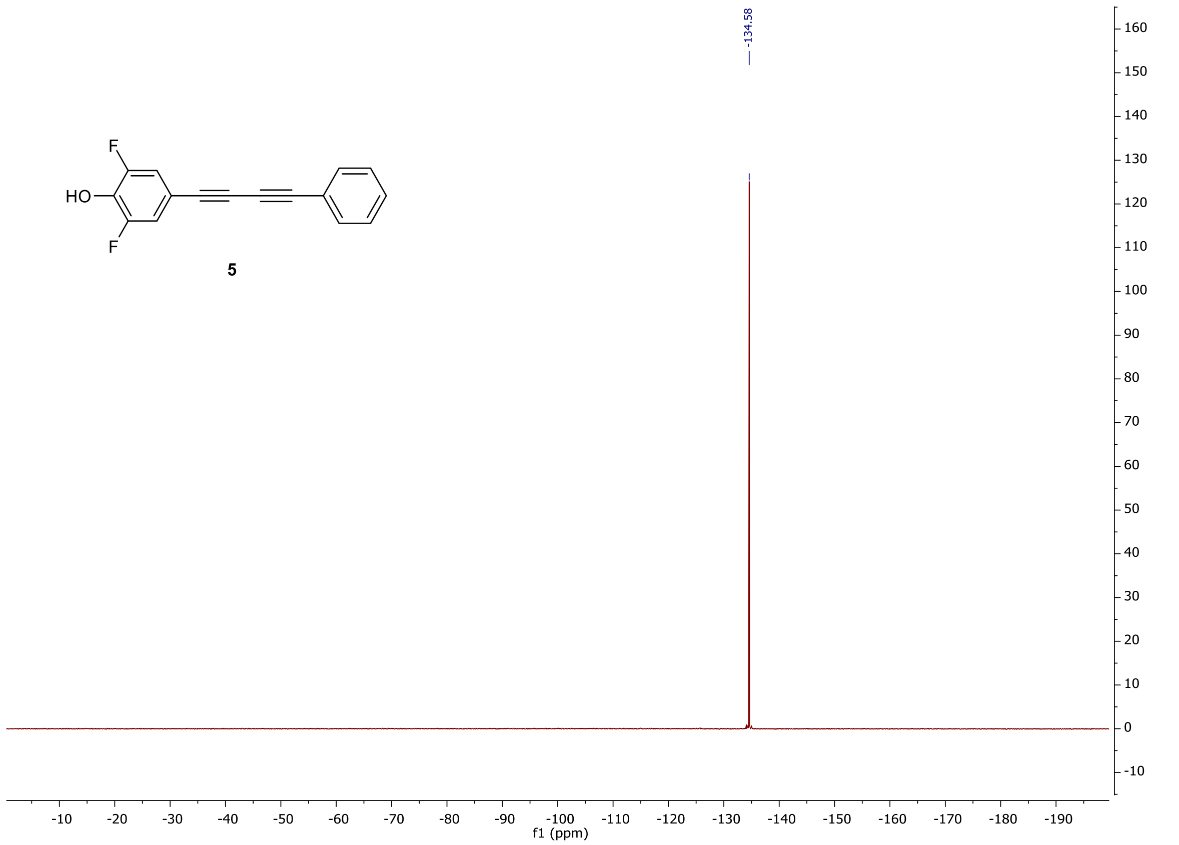

^{13}C NMR spectrum of **5** (101 MHz, $CDCl_3$)

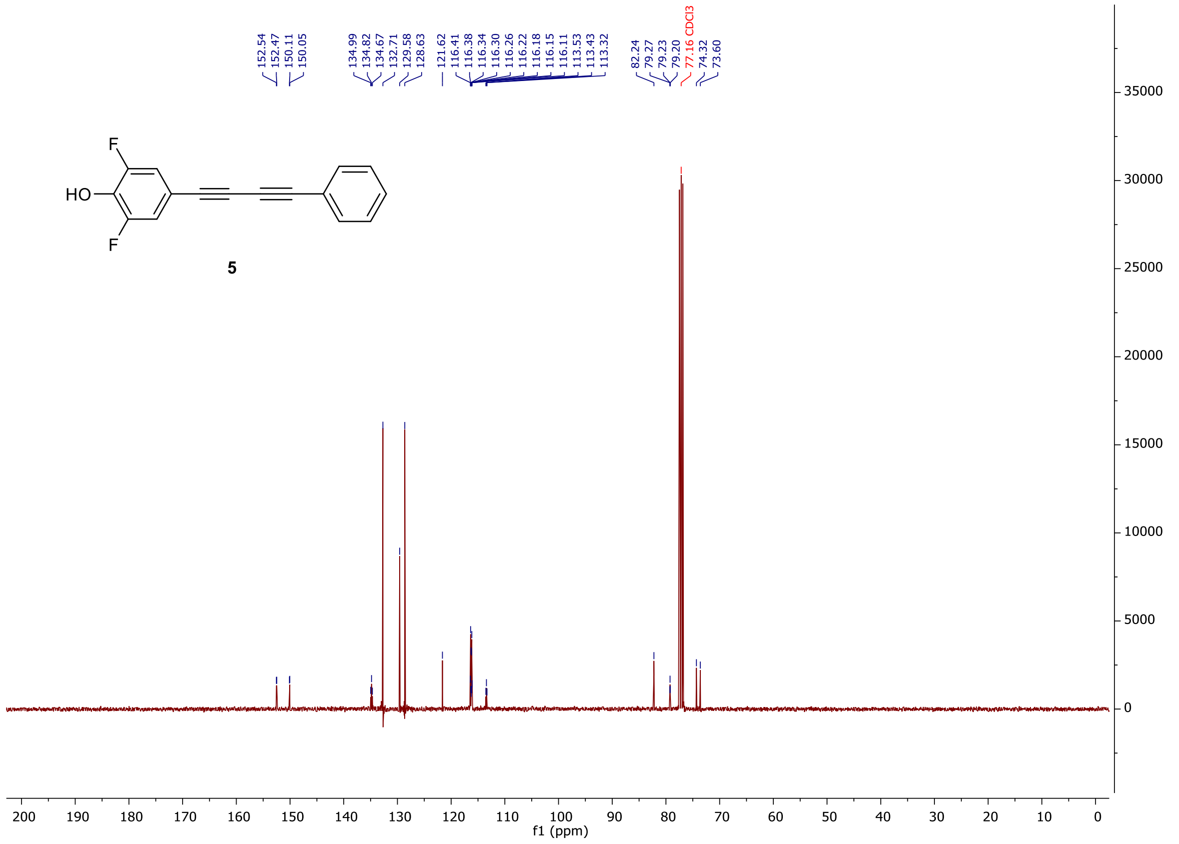

^{1}H NMR spectrum of **4** (400 MHz, $CDCl_3$)

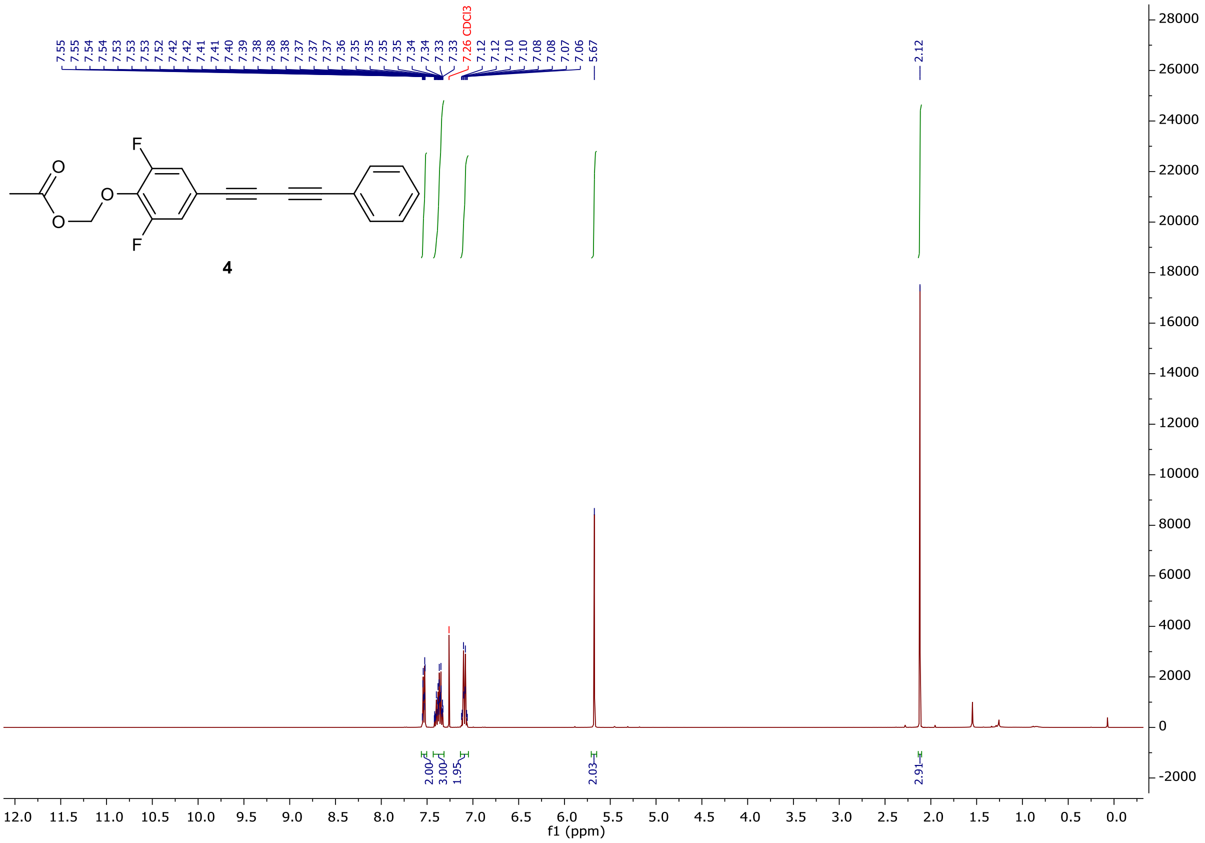

^{19}F NMR spectrum of **4** (376 MHz, $CDCl_3$)

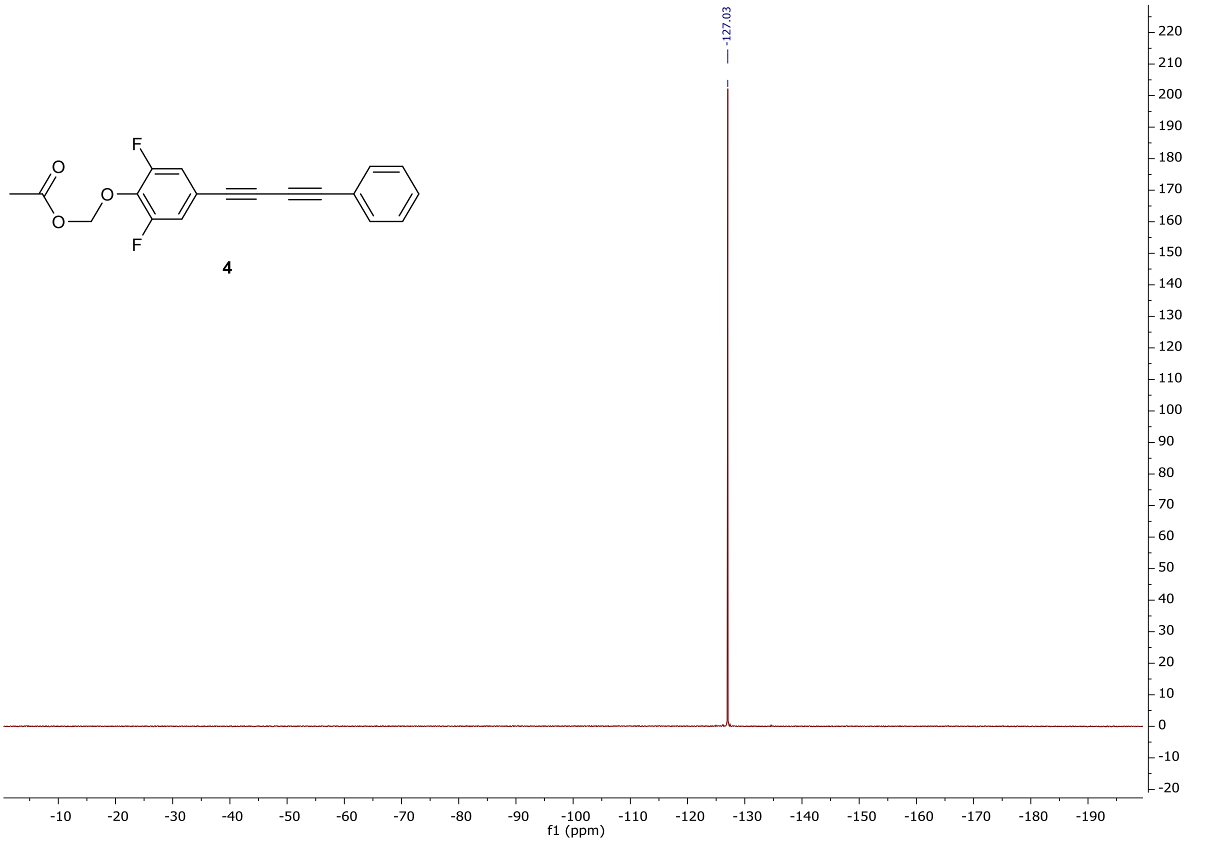

^{13}C NMR spectrum of **4** (101 MHz, $CDCl_3$)

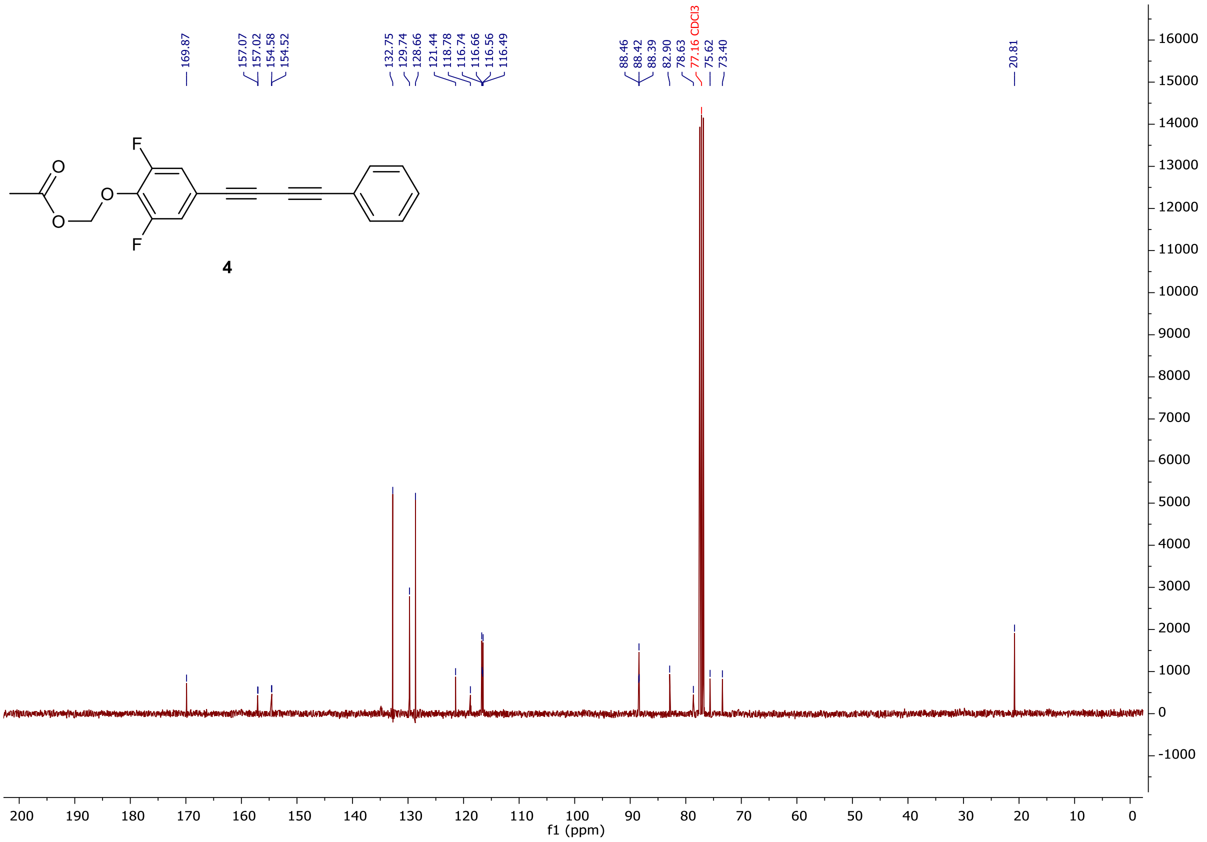

^{1}H NMR spectrum of **6** (400 MHz, $CDCl_3$)

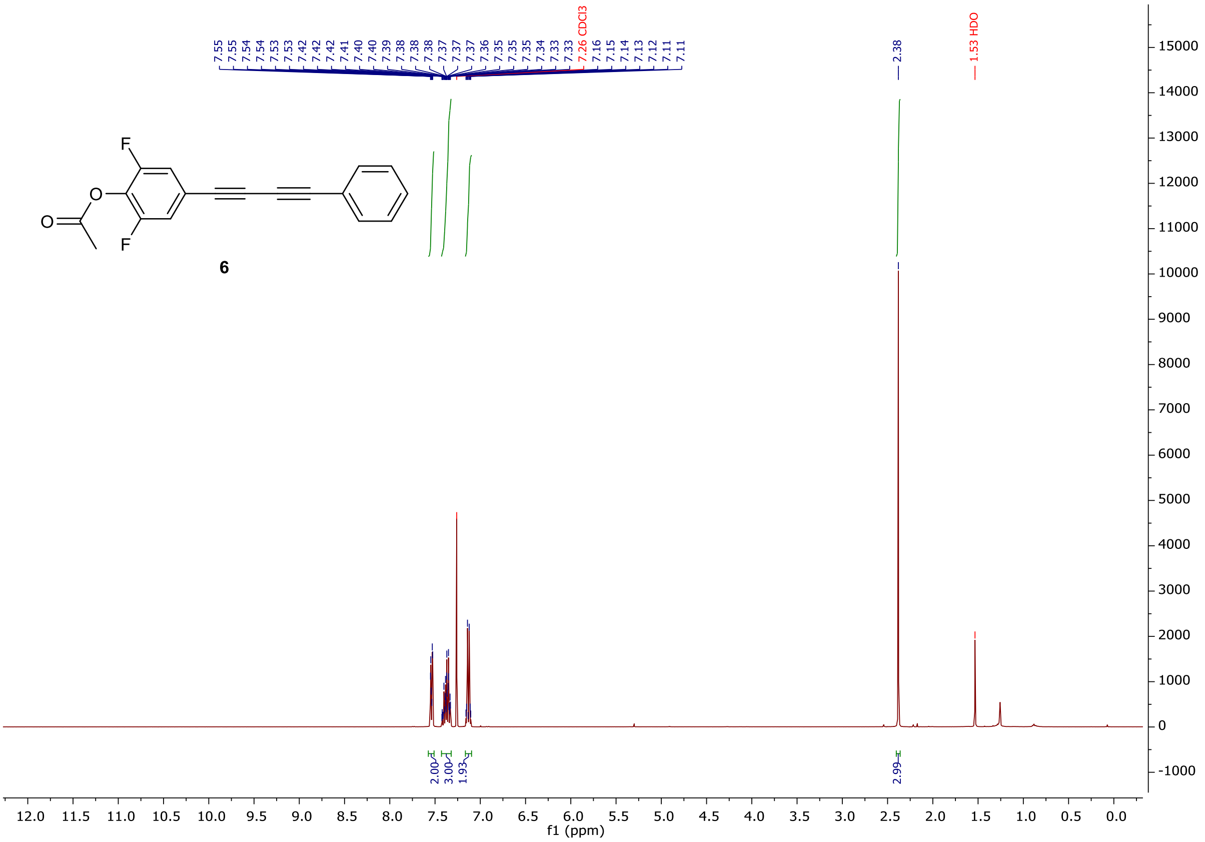

^{19}F NMR spectrum of **6** (376 MHz, $CDCl_3$)

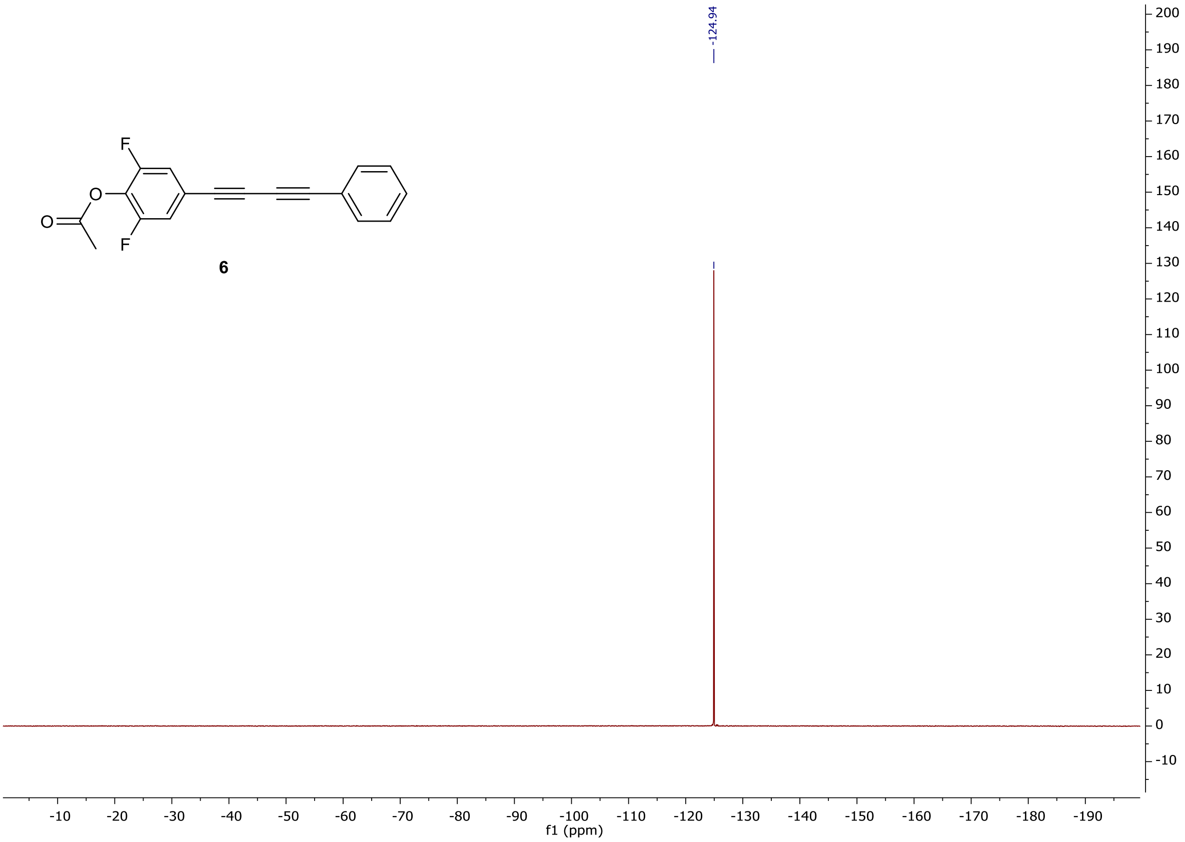

^{13}C NMR spectrum of **6** (101 MHz, $CDCl_3$)

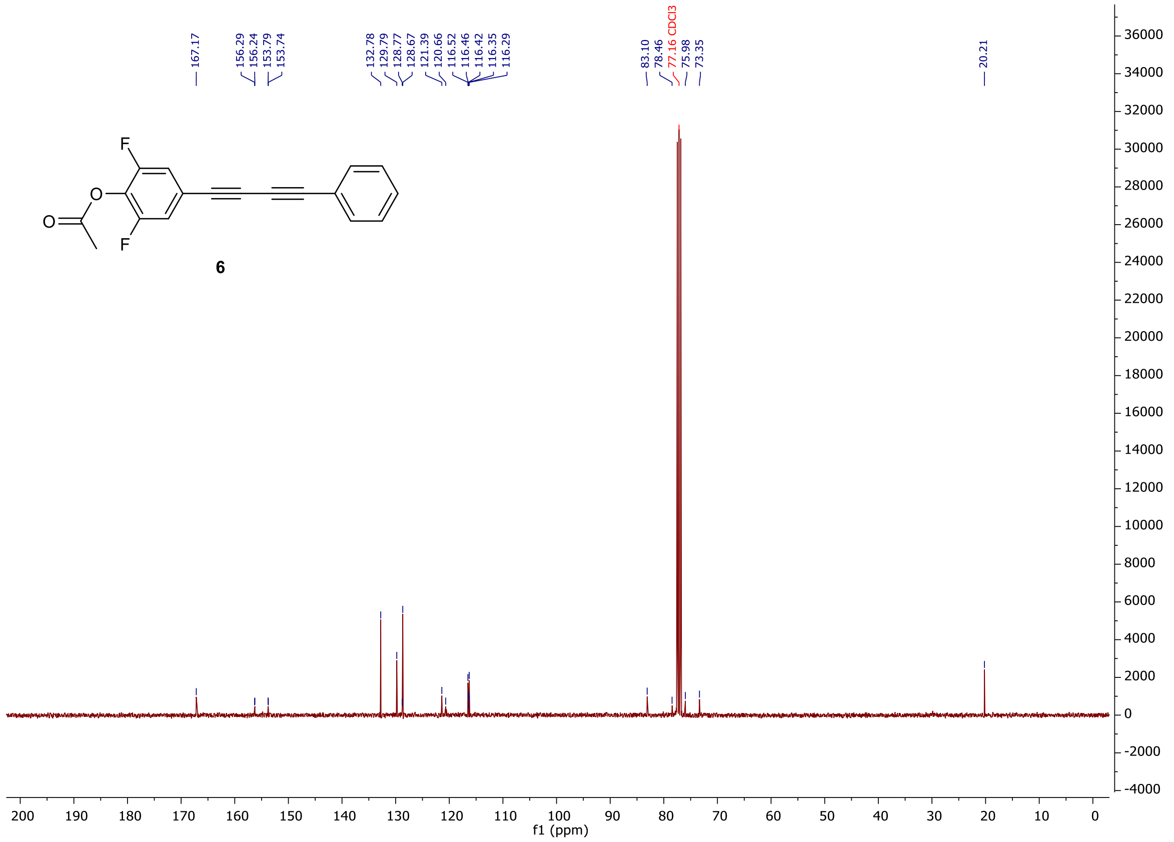

Supplement: Supplementary file 1 — ac2c05708_si_001.pdf [file ac2c05708_si_001.pdf]
